# Supplementary material for: Influence of Sociodemographic, Premorbid, and Injury-Related Factors on Post-Traumatic Stress, Anxiety, and Depression after Traumatic Brain Injury
Source: J Clin Med. 2023 Jun 6;12(12):3873. doi: 10.3390/jcm12123873 (PMC10299140; doi:10.3390/jcm12123873)
Supplement: Supplementary file 1 [file jcm-12-03873-s001.zip › S2_Literary_Review_Additional_Methods_and_Results.pdf]

Influence of Sociodemographic, Premorbid, and Injury-Related Factors on Post-traumatic Stress, Anxiety, and Depression after Traumatic Brain Injury – Online Supplement S2

Fabian Bockhop, Katrin Cunitz, Marina Zeldovich, Anna Buchheim, Tim Beissbarth, York Hagmayer, Nicole von Steinbuechel, and on behalf of the CENTER-TBI Participants and Investigators

Table of Contents

Literary Review 2

Additional Information on Methods and Results 10

- **Additional Information on Methods** 10
  - Regression Models 10
- **Descriptive Analyses** 14
- **Additional Information on Results** 16
  - Regression Models 16
  - Correlations between PROM scores across Psychopathological Domains 18
  - Correlations between Three-Month and Six-Month PROM Scores 18
  - Endorsement of Psychopathological Symptoms 18

Analyses without exclusions 22

References 32

Literary Review

Table S1. Literary review on factors associated with psychopathological outcomes in adult civilians after TBI (adapted from [1]).

Factor	PTSD	Anxiety	Depression
**Age**	**Increased risk of new-onset** *depression* and **PTSD in older adults** [2]<br><br>Anxiety-related disorders (including **PTSD**) were associated with *female sex* and *lower functional recovery*. **No significant effect** of **age**, *employment, marital status*, and length of *LOC* [3]<br><br>**age-group*sex was associated** with six-month **PTSD** in young adults after mild TBI; compared with *females 30-39y., males 18-29y. and males 30-39y* had *decreased PTSD symptomatology* [4]<br><br>**Age at injury**, *gender*, and *years of education* **did not differ** significantly between participants with and without injury-related **PTSD** after moderate to severe TBI [5]<br><br>**Age at injury** and *years of education* had **no significant association** with **PTSD** after TBI [6]	**Significant anxiety** and *depression* i**n patients aged 50 and above** [7]<br><br>*Female gender, lower education, unemployment* were *associated* with *depression* after TBI. *Unemployment* and **older age** were **associated** with **anxiety** after TBI [8] **Anxiety** was associated with *lower functional recovery*. **No significant effect** of **age**, *female sex, employment, marital status*, and length of *LOC* [3]<br><br>**Younger age** was related to **more severe GAD** [9]<br><br>**Middle age**, *lower education*, and *preinjury mental health treatment* were **risk factors** for later **anxiety** after moderate to severe TBI [10]	**Increased risk of new-onset depression** and *PTSD* **in older adults** [2] **Significant** *anxiety* and **depression in patients aged 50 and above** [7]<br><br>**Risk of MDD** after TBI was associated with *history of MDD* prior to injury and **older age** [11]<br><br>*Depression* was associated with *lower functional recovery*. **No significant effect** of **age**, *female sex, employment, marital status*, and length of *LOC* with **depression** after TBI [3]<br><br>**No significant association** of **MDD** with *gender*, **age**, *education level, working status*, or *marital status* after severe TBI [12]
**Sex/Gender**	**Worse outcomes** in **women** than men (*depression*, **PTSD**) [13] **Higher PTSD risk** in **men** compared to women after TBI [14]<br><br>Anxiety-related disorders (including **PTSD**) were associated with **female sex** and *lower functional recovery*. *No significant effect* of *age, employment, marital status*, and length of *LOC* [3]	**Higher risk for anxiety** and *depressive disorders* **after TBI in females**, those *without employment*, and *those with psychiatric history* before TBI [18]<br><br>**Women** reported *more MDD symptoms* and **more GAD symptoms** than men after TBI [19]<br><br>**Anxiety** was associated with *lower functional recovery*. **No significant effect** of *age*, **female**	**Higher risk for** *anxiety* and **depressive disorders after TBI in females**, those *without employment*, and those *with psychiatric history* before TBI [18]<br><br>**Women** reported **more MDD symptoms** and *more GAD symptoms* than men after TBI [19]<br><br>**Female gender**, *lower education, unemployment* were *associated* with **depression** after TBI.

Factor	PTSD	Anxiety	Depression
	A **less favorable PTSD** trajectory was associated with **female gender,** *TBI severity (i.e., mild TBI)*, and *admission to ICU* [15]<br><br>**Female gender**, *LOC* and intentional *injury cause* (e.g., assault) predicted **PTSD** after TBI [16]<br><br>**No significant TBI:sex interaction** for **PTSD symptoms**, nor *anxiety* and *depression* [17]<br><br>**age-group*sex was associated** with six-month **PTSD** in young adults after mild TBI; compared with *females 30-39y., males 18-29y. and males 30-39y* had *decreased PTSD symptomatology* [4]<br><br>*Age at injury*, **gender**, and *years of education* **did not differ** significantly between participants with and without injury-related **PTSD** after moderate to severe TBI [5]	**sex**, *employment, marital status*, and length of *LOC* [3]<br><br>**No significant TBI:sex interaction** for *PTSD symptoms*, nor for **anxiety** and *depression* [17]	*Unemployment* and *older age* were associated with *anxiety* after TBI [8]<br><br>**Higher depression** after TBI was associated with *more severe TBI* and **female gender**. *Depression severity* was associated with *injury cause* [20]<br><br>**No significant association** of **MDD** with **gender**, *age, education level, working status*, or *marital status* after severe TBI [12]<br><br>**No significant TBI:sex interaction** for *PTSD symptoms*, nor *anxiety* and **depression** [17]<br><br>*Depression* was associated with *lower functional recovery*. **No significant effect** of *age*, **female sex**, *employment, marital status*, and length of *LOC* [3]
**Education**	Association of **lower educational level** with greater *depression*, *anxiety*, **PTSD** severity [17]<br><br>**PTSD** after TBI was associated with **lower education,** *premorbid mental disorder*, and *injury cause* (i.e., assault, violence). *No significant effect* of duration of *LOC* [21]<br><br>**Reduced odds** for **PTSD** screening after TBI were associated with **more years of education** and in *married* individuals. *Higher odds* for *PTSD* screening after TBI were associated with *prior psychiatric history* and *TBI cause* [22]<br><br>*Age at injury*, *gender*, and **years of education did not differ** significantly between	Association of **lower educational level** with greater *depression*, **anxiety**, *PTSD* severity [17]<br><br>*Middle age*, **lower education**, and *preinjury mental health treatment* were **risk factors** for later **anxiety** after moderate to severe TBI [10]<br><br>Significant associations of **lower education** with higher **anxiety** and *depression* after TBI rarely reported (**3/20 studies** in review) [18]	Association of **lower educational level** with greater **depression**, *anxiety*, *PTSD* severity [17]<br><br>*Female gender*, **lower education**, *unemployment* were *associated* with **depression** after TBI. *Unemployment* and *older age* were associated with *anxiety* after TBI [8]<br><br>Significant associations of **lower education** with higher *anxiety* and **depression** after TBI rarely reported (**3/20 studies** in review) [18]<br><br>**No significant association** of **MDD** with *gender*, *age*, **education level**, *working status*, or *marital status* after severe TBI [12]

Factor	PTSD	Anxiety	Depression
	participants with and without injury-related **PTSD** after moderate to severe TBI [5]<br><br>*Age at injury* and **years of education** had **no significant association** with **PTSD** after TBI [6]		**MDD** after TBI was associated with **lower education** and *premorbid mental disorder. No significant effect* of *injury cause* (i.e., assault, violence) and duration of *LOC* [21]
**Employment**	Anxiety-related disorders (including **PTSD**) were associated with *female sex* and *lower functional recovery*. **No significant effect** of *age*, **employment**, *marital status*, and length of *LOC* [3]	**Anxiety** was associated with *lower functional recovery*. **No significant effect** of *age*, *female sex*, **employment**, *marital status*, and length of *LOC* [3]<br><br>*Female gender, lower education, unemployment* were *associated* with *depression* after TBI. **Unemployment** and *older age* were associated with **anxiety** after TBI [8]<br><br>**Higher risk for anxiety** and *depressive disorders* after TBI in *females*, those **without employment**, and those *with psychiatric history before TBI* [18]<br><br>**Stable employment** was **associated** with **no anxiety** or *depression* after TBI [23]	*Depression* was associated with *lower functional recovery*. **No significant effect** of *age*, *female sex*, **employment**, *marital status*, and length of *LOC* with **depression** after TBI [3]<br><br>*Female gender, lower education,* **unemployment** were *associated* with **depression** after TBI. *Unemployment* and *older age* were associated with *anxiety* after TBI [8]<br><br>**Higher risk for** *anxiety* and **depressive** *disorders* after TBI in *females*, those **without employment**, and those *with psychiatric history before TBI* [18]<br><br>**Stable employment** was **associated** with **no** *anxiety* or **depression** after TBI [23]<br><br>**No significant association** of **MDD** with *gender, age, education level*, **working status**, or *marital status* after severe TBI [12]

Factor	PTSD	Anxiety	Depression
**Living situation/**<br><br>**Marital status**	Anxiety-related disorders (including **PTSD**) were associated with *female sex* and *lower functional recovery*. **No significant effect** of *age, employment*, **marital status**, and length of *LOC* [3]<br><br>**Living alone** was an independent **predictor** of *depression* and/or **PTSD** after TBI. *Depression* and *PTSD* were associated with a significantly *decreased functional outcome* [24]<br><br>**Reduced odds** for **PTSD** screening after TBI were associated with *more years of education* and in **married** individuals. *Higher odds* for *PTSD* screening after TBI were associated with *prior psychiatric history* and *TBI cause* [22]	**Anxiety** was associated with *lower functional recovery*. **No significant effect** of *age, female sex, employment*, **marital status**, and length of *LOC* [3]	*Depression* was associated with *lower functional recovery*. **No significant effect** of *age, female sex, employment*, **marital status**, and length of *LOC* with **depression** after TBI [3]<br><br>**Living alone** was an independent **predictor** of **depression** and/or *PTSD* after TBI. *Depression* and *PTSD* were associated with a significantly *decreased functional outcome* [24]<br><br>Participants that were either **single** or **no longer married** had **worse depression** prognoses [25]<br><br>**No significant association** of **MDD** with *gender, age, education level, working status*, or **marital status** after severe TBI [12]
**Premorbid psychological problems**	**PTSD** after TBI was associated with *lower education*, **premorbid mental disorder**, and *injury cause* (i.e., assault, violence). *No significant effect* of duration of *LOC* [21]<br><br>*Reduced odds* for *PTSD* screening after TBI were associated with *more years of education* and in *married* individuals. **Higher odd**s for **PTSD** screening after TBI were associated with **prior psychiatric history** and *TBI cause* [22]<br><br>The most important risk factor **associated** with *depression* and **PTSD** was lifetime **history** of any **psychiatric disorder** [26]	**Pre-injury mental health treatment** as a risk factor for developing higher and more persistent levels of **anxiety** during 10 years after TBI [27]<br><br>**Higher risk for anxiety** and *depressive disorder*s after TBI in *females*, those *without employment,* and those **with psychiatric history before TBI** [18]<br><br>*Middle age, lower education* and **preinjury mental health treatment** were **risk factors** for later **anxiety** after moderate to severe TBI [10]	**MDD** after TBI was associated with *lower education* and **premorbid mental disorder**. *No significant effect* of *injury cause* (i.e., assault, violence) and duration of *LOC* [21] **Higher risk for** *anxiety* and **depressive disorders after TBI** in *females*, those *without employment*, and **those with psychiatric history before TBI** [18]<br><br>Risk of **MDD** after TBI **was associated** with **history of MDD** prior to injury and *older age* [11]<br><br>The most important risk factor **associated** with **depression** and *PTSD* was lifetime **history** of any **psychiatric disorder** [26]

Factor	PTSD	Anxiety	Depression
**TBI cause**	**Road traffic accidents and other injury causes (vs falls)** were associated with **greater PTSD symptom severity** and *higher anxiety* [17]<br><br>**PTSD** after TBI was associated with *lower education, premorbid mental disorder*, and **injury cause** (i.e., assault, violence). *No significant effect* of duration of *LOC* [21]<br><br>*Female gender*, *LOC* and intentional **injury cause** (e.g., assault) predicted **PTSD** after TBI [16]<br><br>*Reduced odds* for *PTSD* screening after TBI were associated with *more years of education* and in *married* individuals. **Higher odd***s* for **PTSD** screening after TBI were associated with *prior psychiatric history* and **TBI cause** [22]<br><br>**Injury cause** (i.e., assault) significantly associated with **PTSD** scores after TBI [28]	**Road traffic accidents and other injury causes (vs falls)** were associated with *greater PTSD symptom severity* and **higher anxiety** [17]<br><br>The *relationship* between *injury severity* and **anxiety** after TBI was **moderated** by **TBI cause** [10]	*Higher depression* after TBI was associated with *more severe TBI* and *female gender*. **Depression severity** was associated with **injury cause** [20]<br><br>**MDD** after TBI was associated with *lower education* and *premorbid mental disorder*. **No significant effect** of **injury cause** (i.e., assault, violence) and duration of *LOC* [21]
**TBI severity**	**No clear effect** of **TBI severity** on development of the **PTSD** [29]<br><br>*Anxiety disorders* were *more likely* to develop in those with *mild TBI*. **PTSD** has been reported to be associated with TBI, **regardless of injury severity** [30]<br><br>The evidence for an association of an *increased severity of the TBI* with an *increased risk* of *psychiatric disorders* was *mixed* for all disorders (including **GAD** and *MDD*), except for **PTSD** (i.e., **increased risk of PTSD** with **milder TBI**) [31]	Increased **anxiety** and *depression* in *females* and patients with **more severe TBI** [33]<br><br>**Anxiety disorders** were **more likely** to develop in those with **mild TBI**. *PTSD* has been reported to be associated with TBI, *regardless of injury severity* [30]<br><br>The evidence for an association of an **increased severity of the TBI** with an **increased risk** of **psychiatric disorders** was **mixed** for all disorders (including **GAD** and *MDD*), except for *PTSD* (i.e., *increased risk of PTSD* with *milder TBI*) [31]	Increased *anxiety* and **depression** in *females* and patients with **more severe TBI** [33]<br><br>The evidence for an association of an **increased severity of the TBI** with an **increased risk** of **psychiatric disorders** was **mixed** for all disorders (including *GAD* and **MDD**), except for *PTSD* (i.e., *increased risk of PTSD* with *milder TBI*) [31]<br><br>**Higher depression** after TBI was associated with **more severe TBI** and *female gender*. *Depression severity* was associated with *injury cause* [20]

**Factor**	**PTSD**	**Anxiety**	**Depression**
	**TBI severity** was *related* to the degree of *depression* and *anxiety*, but **unrelated** to **PTSD** [32].<br><br>A **less favorable PTSD** trajectory was associated with *female gender*, **TBI severity** (mild TBI), and *admission to ICU* [15]	**TBI severity** was **related** to the degree of *depression* and **anxiety**, but *unrelated* to *PTSD* [32].<br><br>The **relationship** between **injury severity** and **anxiety** after TBI was *moderated* by *TBI cause* [10]	**TBI severity** was **related** to the degree of **depression** and *anxiety*, but *unrelated* to *PTSD* [32].
**LOC**	Anxiety-related disorders (including **PTSD**) were associated with *female sex* and *lower functional recovery*. **No significant effect** of *age, employment, marital status*, and length of **LOC** [3]<br><br>*Female gender*, **LOC,** and intentional *injury cause* (e.g., assault) predicted **PTSD** after TBI [16]<br><br>**PTSD** after TBI was associated with *lower education***,** *premorbid mental disorder*, and *injury cause* (i.e., assault, violence). **No significant effect** of duration of **LOC** [21]<br><br>**Debatable** effect of **LOC** in the relationship of **PTSD** and TBI [34]	**Anxiety** was associated with *lower functional recovery*. **No significant effect** of *age, female sex, employment, marital status*, and length of **LOC** [3]	*Depression* was associated with *lower functional recovery*. **No significant effect** of *age, female sex, employment, marital status*, and length of **LOC** with **depression** after TBI [3]<br><br>**MDD** after TBI was associated with *lower education* and *premorbid mental disorder*. **No significant effect** of *injury cause* (i.e., assault, violence) and duration of **LOC** [21]
**Extracranial injuries**	There was a **significant negative association** between **extracranial injuries** and **PTSD symptoms** [35].<br><br>Patients suffering from **extracranial injuries** after TBI reported significantly **worse PTSD** and *MDD* symptoms [28]<br><br>Presence of **phyiscal injuries increases** the risk of developing of **PTSD** after TBI [36]	Higher risk for *more severe MDD* and **GAD** for those being *more severely disabled*, **having experienced major extracranial injuries**, *an intensive care unit stay*, and being *female* [9]	Higher risk for **more MDD** and *GAD* for those being *more severely disabled*, **having experienced major extracranial injuries**, *an intensive care unit stay*, and being *female* [9]<br><br>Patients suffering from **extracranial injuries** after TBI reported significantly **worse** *PTSD* and **MDD** symptoms [28]<br><br>Everyday functioning including **depression** was influenced by concomitant **extracranial injuries** after mild TBI [38]

Factor	PTSD	Anxiety	Depression
	There was **no relationship** of **extra-cranial injury severity** with *MDD* or **PTSD** after mild to moderate TBI [37]		There was **no relationship** of **extra-cranial injury severity** with **MDD** or *PTSD* after mild to moderate TBI [37]
**Clinical care pathways**	A **less favorable PTSD** trajectory was associated with *female gender*, *TBI severity* (mild TBI), and **admission to ICU** [15]<br><br>**Higher risk** to develop **PTSD** for injury patients (including TBI) admitted to an **ICU** [39]	Higher risk for **more severe** *MDD* and **GAD** for those being *more severely disabled*, having experienced *major extracranial injuries*, **an ICU stay**, and being *female* [9]	Higher risk for **more MDD** and *GAD* for those being *more severely disabled*, having experienced *major extracranial injuries*, **an ICU stay**, and being *female* [9]
**Functional recovery**	Anxiety-related disorders (including **PTSD**) were associated with *female sex* and **lower functional recovery**. *No significant effect* of *age, employment, marital status*, and length of *LOC* [3]<br><br>**Lower functional status** was associated with **greater** odds of **PTSD** after moderate to severe TBI [5]<br><br>*Living alone* was an independent *predictor* of *depression* and/or *PTSD* after TBI. *Depression* and **PTSD** were associated with a significantly **decreased functional outcome** [24]<br><br>**Inconclusive results** on the relationship between psychiatric conditions such as *depression* and **PTSD** with **(long-term) outcome** and **recovery** after mild TBI [40]	**Anxiety** was associated with **lower functional recovery**. *No significant effect* of *age, female sex, employment, marital status*, and length of *LOC* [3]<br><br>Changes in **functional status** predicted *depression* and **anxiety** after TBI [41]<br><br>Participants showing **poorer functional outcome** showed **higher levels of anxiety** [42]	**Depression** was associated with **lower functional recovery**. *No significant effect* of *age, female sex, employment, marital status*, and length of *LOC* with *depression* after TBI [3]<br><br>Changes in **functional status** predicted **depression** and *anxiety* after TBI [41]<br><br>*Living alone* was an independent *predictor* of *depression* and/or *PTSD* after TBI. **Depression** and *PTSD* were associated with a significantly **decreased functional outcome** [24]<br><br>**Inconclusive results** on the relationship between psychiatric conditions such as **depression** and *PTSD* with **(long-term) outcome** and **recovery** after mild TBI [40]

Note. TBI = traumatic brain injury, PTSD = Post-traumatic Stress Disorder, GAD = Generalized Anxiety Disorder, MDD = Major Depressive Disorder, ISS = total injury severity score, ICU = intensive care unit, LOC = loss of consciousness; **bold** entries = association between respective factor and the psychopathological outcome, *italic* entries = association between respective factors and other psychopathological outcomes.

Additional Information on Methods and Results

Additional Information on Methods

Regression Models

Analyzing Psychopathological Screening Diagnoses. As a first step, binomial logistic regression (LR) models are employed, which have been frequently used to model outcomes after TBI [1,2]. Screening diagnoses (PTSD/GAD/MDD present vs. absent) based on the respective aforementioned clinical cutoffs (i.e., PCL-5 ≥ 31; GAD-7 ≥ 10; PHQ-9 ≥ 10) serve as dependent variables that are tested with regard to the influence of the aforementioned sociodemographic, premorbid, and injury-related factors as well as previously proposed interactions (i.e., sex:age, sex:LOC) [3]. Specifically, the LR models predict the probability of above-threshold levels of psychopathology. The overall model fit was assessed using Nagelkerke's R^2 [4] and area under the curve (AUC) [5]. Nagelkerke's R^2 can range from 0—1, with higher values indicating better model fit, and suggests the superior fit of the estimated model relative to an intercept-only null model. The AUC is used to evaluate the ability of the model to identify individuals above vs. below the clinical screening thresholds with regard to PTSD, GAD, and MDD. On the factor level, multicollinearity was examined based on the variance inflation factor (VIF) [6] and all VIFs were below the proposed cutoff of ten, indicating the absence of pronounced multicollinearity. Finally, OR with 95%-confidence intervals (*CI*) were calculated as an indicator of the association of the respective factor with the model outcome.

Upon further exploration, we found substantial overdispersion in all PROM scores, indicating that the observed variances exceeded the respective means. Additionally, the residuals for the average PROM scores were non-normally distributed, as evidenced by the observation of histograms and QQ-plots alongside Shapiro-Wilk tests ($p<.001$; see Figure S1, A—C). Therefore, we concluded that central assumptions for linear regression analyses were violated and the use of alternative non-linear models based on Negative Binomial (NB) distributions was more appropriate [7,8].

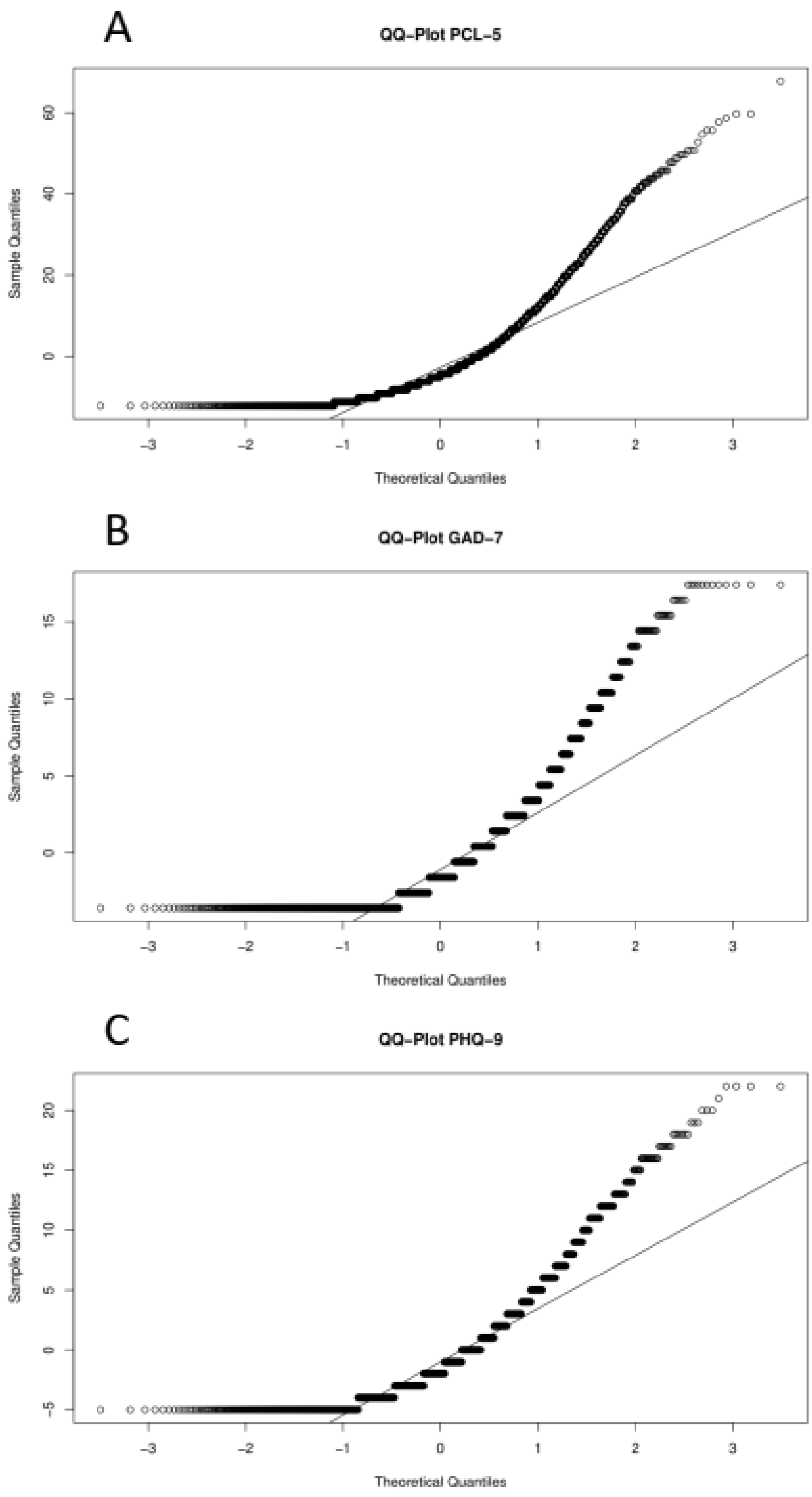

Figure S1. PCL-5, Posttraumatic Stress Disorder Checklist for DSM-5; GAD-7, Generalized Anxiety Disorder Scale 7; PHQ-9, Patient Health Questionnaire 9. The plots show the distribution of residuals for the PCL-5 score (A), the GAD-7 score (B), and the PHQ-9 score (C). Deviations from line indicate non-normal distribution.

Analyzing Count Data. Non-linear NB models are used to analyze non-negative count data [9] which can serve as targets in health research (e.g., number of clinical symptoms, days of hospitalization). For the current study, individuals' PROM scores were transposed to reflect the endorsement of clinical symptoms related to PTSD, GAD, or MDD in correspondence to the respective diagnostic criteria [10–12]. Based on previous recommendations, the response categories 2 (*moderately bothered*) to 4 (*extremely bothered*) in the PCL-5 [13] and 1 (*bothered on several days*) to 3 (*bothered nearly every day*) in the GAD-7 and the PHQ-9 [14] were collapsed. This resulted in the dichotomized coding of symptom endorsement (0 = symptom absent, 1 = symptom present) which was summarized into symptom counts for PTSD (0-20), GAD (0-7), and MDD (0-9). The respective distribution of these newly obtained count data was inspected using histograms (see Figure S2, A—C) and revealed a substantial proportion of individuals who did not indicate any clinical symptoms of PTSD (35.81%), GAD (33.40%), or MDD (19.86%).

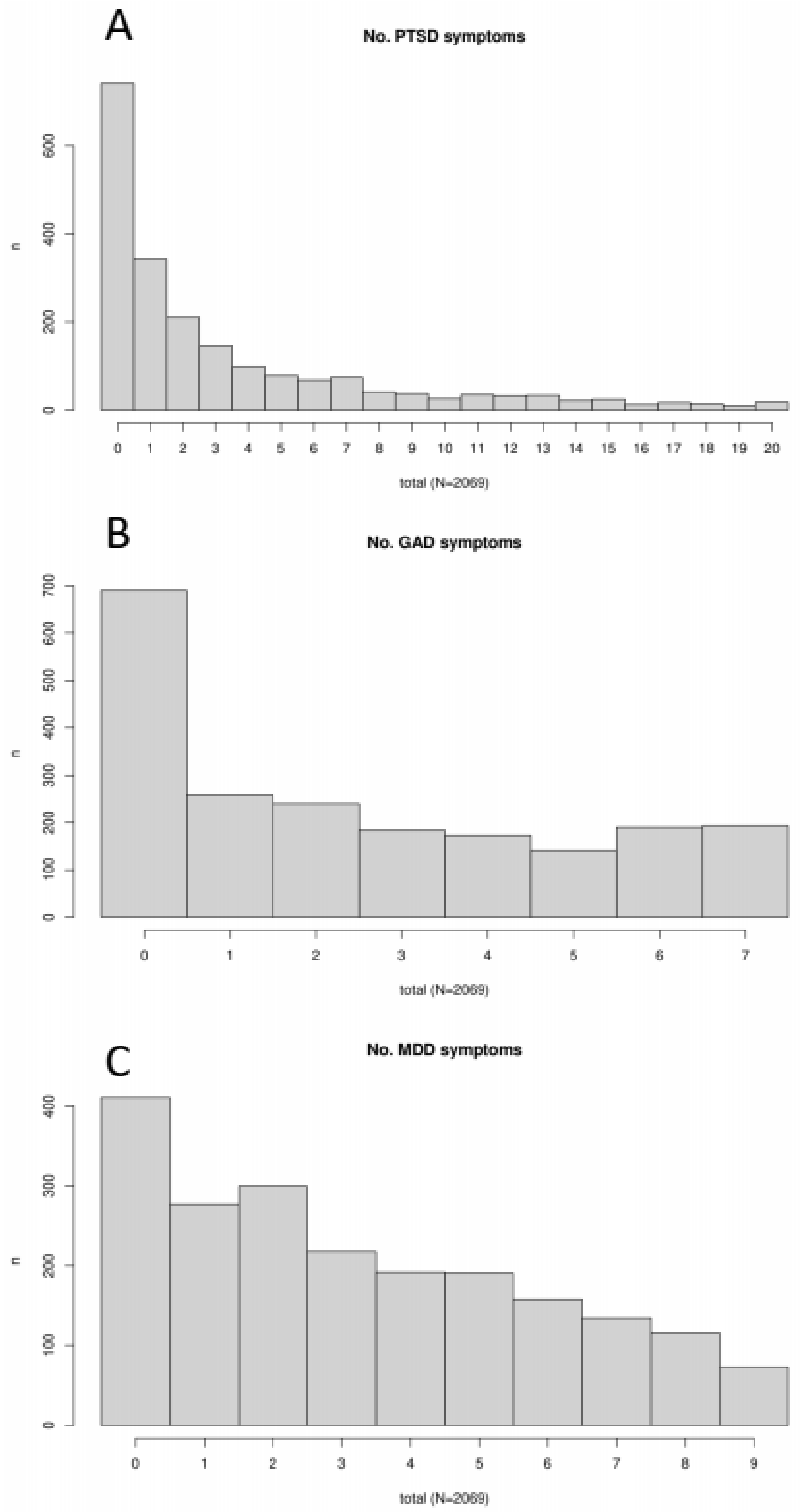

Figure S2. PTSD, Posttraumatic Stress Disorder; GAD, Generalized Anxiety Disorder; MDD, Major Depressive Disorder. The graphs show the distribution of symptoms of PTSD (A), GAD (B), and MDD (C) across the total sample. The size of bar graph indicates the number of cases.

Occurrence and Frequency of Psychopathological Symptoms. In order to account for these subgroups of individuals who did not experience psychopathological symptoms while also controlling for overdispersion and non-normally distributed residuals, the subsequent regression analyses employed univariate zero-inflated negative binomial regressions (ZINB) [3,15–17]. ZINB models assumed an underlying NB distribution in the data and comprised (1) a *zero*-part that estimated which factors were associated with the occurrence or absence of symptoms associated with PTSD, GAD, or MDD, and (2) a *count*-part focused on non-zero values that analyzed the effect of the factors on the frequency of symptoms associated with PTSD, GAD, or MDD. By employing maximum likelihood estimations, this method enabled the detection of factors that distinctly contributed to the occurrence and/or the frequency of psychopathological symptoms after TBI. For more details, see Lambert (1999) [18].

Three univariate ZINB models were fitted which each targeted another psychopathological symptom count (PTSD, GAD, MDD) as the respective outcome variable. The factors included in these models comprised the aforementioned sociodemographic, premorbid, and injury-related factors. Multicollinearity between factors was examined based on the calculation of the VIF [6]. All VIFs were below the proposed cutoff of 10, indicating the absence of pronounced multicollinearity. The goodness-of-fit for all univariate ZINB models was evaluated by means of likelihood ratio tests with the log-likelihood (LogLik) serving as the statistical readout. Full-null model comparisons were carried out, where significant results ($p<.05$) would indicate superior fit of the respective regression model over the intercept-only model. Finally, the association of the individual factors with the respective model outcomes were evaluated based on OR with 95%-CI for the zero-part and rate ratios (*RR*) with 95%-CI in the count-part of the ZINB models. were calculated as an indicator of the association of the respective factor with the model outcome.

Intensity of Psychopathology. In addition to the models on the psychopathological symptoms, we investigated the effect of the aforementioned sociodemographic, premorbid, and injury-related factors on the intensity of individuals' psychopathological screenings using standard NB models [9,15]. Three univariate NB models were estimated with each PROM score (i.e., PCL-5, GAD-7, PHQ-9) serving as the respective outcome. These models included all aforementioned factors as well as previously proposed interactions (i.e., sex:age, sex:LOC) [3]. The corresponding model fit was estimated using likelihood ratio tests. Full-null model comparisons based on the log-likelihood (LogLik) value with statistical testing were conducted, where significant results ($p<.05$) would indicate that the tested model would outperform an intercept-only null model with regard to data description. Finally, the associations of the individual factors with the respective model outcomes were evaluated based on RR with 95%-CI.

Descriptive Analyses

Descriptive statistics were calculated for the sociodemographic, premorbid, injury-related, and psychopathological characteristics and include information on the sample size, missing data, counts, mean, standard deviation, skewness (*SK*), and kurtosis (*KU*). Preparatory data inspection aimed to identify potentially confounding inherent differences across

language groups with regard to psychopathological outcomes. Descriptive analyses revealed values of *SK* and *KU* outside of the acceptable ranges (i.e., *SK*=±1; *KU*=±2) [19] in all PROM scores (PCL-5: *SK*=1.67, *KU*=5.74; GAD-7: *SK*=1.65, *KU*=5.48; PHQ-9: *SK*=1.42, *KU*=4.76), suggesting that data distributions were largely skewed and asymmetrical. Hence, statistical comparisons of the PCL-5, GAD-7, and PHQ-9 total scores across language groups were conducted by applying non-parametrical Kruskal-Wallis tests alongside Bonferroni-corrected post-hoc Mann-Whitney U-tests suited for non-normally distributed data. Overall, we found significant differences between language subsamples in PCL-5 scores ($H(15)=56.48$, $p<.001$), GAD-7 scores ($H(15)=78.45$, $p<.001$), and PHQ-9 scores ($H(15)=37.48$, $p=.001$). Post-hoc tests revealed few significant differences with small to moderate effects ranging from 0.14 (PCL-5: Dutch—Italian) to 0.36 (PCL-5: French—Latvian). This indicated a reasonably low risk of confounding effects between the language groups. Therefore, no further exclusions were made from the total sample in the main analyses.

Additional Information on Results

Regression Models

The goodness-of-fit of ZINB models was examined using rootograms (see Figure S3, A—C). These rootograms revealed good model fit for PTSD symptoms. The model fit for symptoms of GAD and MDD overall was satisfactory with a tendency for over- or underfit particularly on the extreme ends of the count distribution. All ZINB models were superior to the respective intercept-only null models (LogLik<.001, *df*=47). Moreover, rootograms were compiled also to examine the goodness-of-fit of NB models on PROM scores, including the excess zeros (see Figure S3, D—F). Overall satisfactory fit was found with the majority of estimated deviations being within the threshold of ±2. A slight underestimation of zero values was observed for PCL-5 scores. This was expected since NB models are used to handle overdispersion but are not tailored to estimate excess zeros. Null-model comparisons showed that all NB models outperformed the respective intercept-only models at (LogLik<.001, *df*=24).

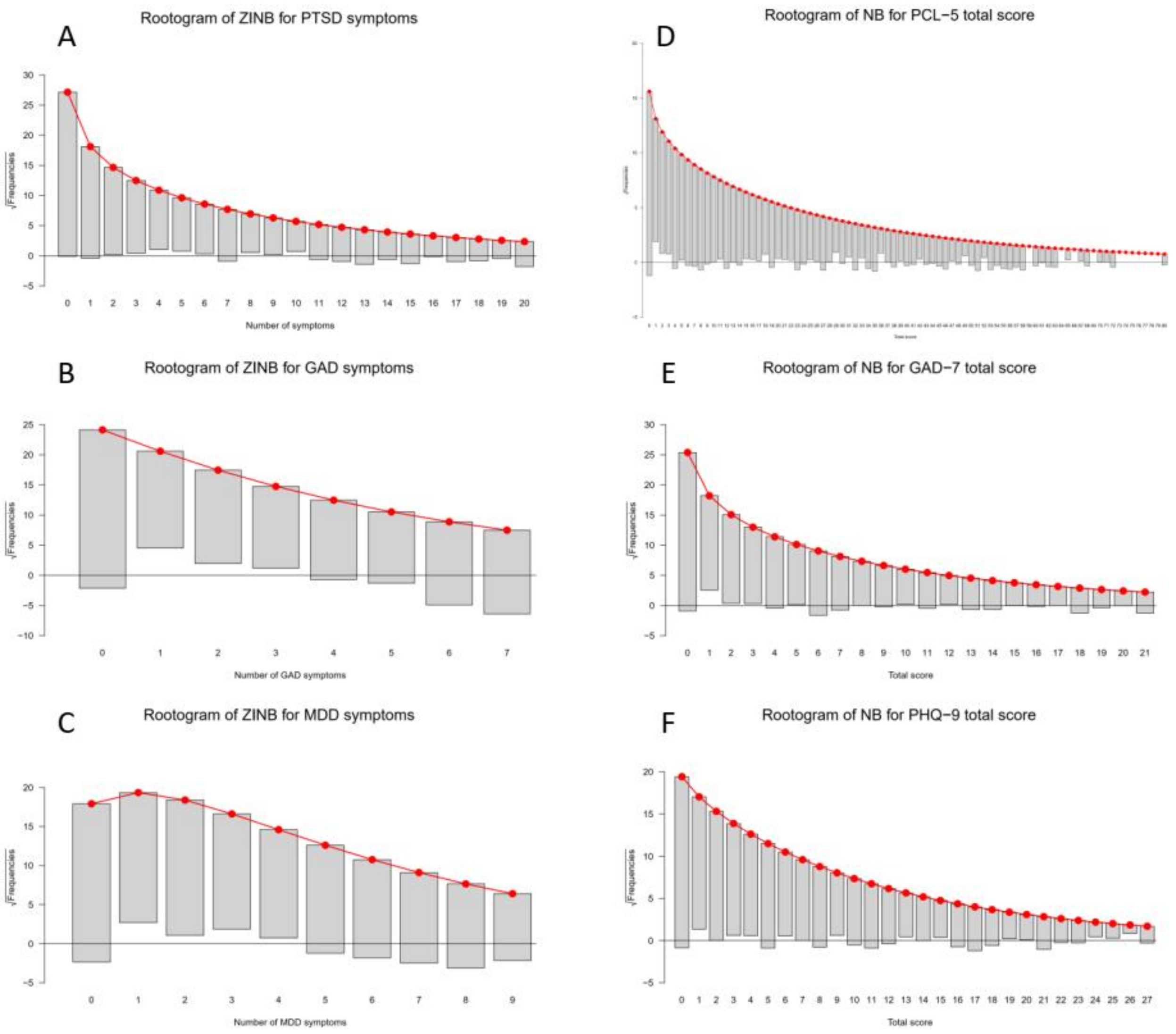

Figure S3. ZINB, Zero-Inflated Negative Binomial Model; PTSD, Posttraumatic Stress Disorder; GAD, Generalized Anxiety Disorder; NB, Negative Binomial Model; MDD, Major Depressive Disorder; PCL-5, Posttraumatic Stress Disorder Checklist for DSM-5; GAD-7, Generalized Anxiety Disorder Scale 7; PHQ-9, Patient Health Questionnaire 9. The Goodness-of-fit (observed and expected counts on a square-root scale) of the ZINBs for symptoms of PTSD (A), GAD (B), and MDD (C), as well as the NBs for PCL-5 scores (D), GAD-7 scores (E), and PHQ-9 scores (F). Frequencies exceeding ± 2 indicate highly over- or underestimated deviations between observed and expected counts.

Correlations between PROM scores across Psychopathological Domains

Individuals in the study sample indicated an average PCL-5 total score of 12.25 (*SD*=13.64), a GAD-7 total score of 3.59 (*SD*=4.49), and a PHQ-9 total score of 5.00 (*SD*=5.30) at six months post TBI. All paired Spearman rank correlations were significant ($p<.001$) with large effect sizes (ϱ=0.71–0.77; see Table S2) [20].

Table S2. Correlational Analyses for psychopathological PROMs at three and six months after TBI

	GAD-7 (6M)	PHQ-9 (6M)	PCL-5 (3M)	GAD-7 (3M)	PHQ-9 (3M)
PCL-5 (6M)	0.707	0.725	0.730	-	-
GAD-7 (6M)	-	0.768	-	0.691	-
PHQ-9 (6M)	-	-	-	-	0.725

Note. PCL-5, Posttraumatic Stress Disorder Checklist for DSM-5; GAD-7, Generalized Anxiety Disorder Scale 7; PHQ-9, Patient Health Questionnaire 9; 6M, six-months assessment; 3M, three-months assessment. Correlations were analyzed using Spearman rank correlations with respective effect sizes (ϱ). All correlations were significant ($p<.001$).

Correlations between Three-Month and Six-Month PROM Scores

The majority of individuals in the final sample had also completed three-month assessments with the PCL-5 (*n*=1630, 78.78%), the GAD-7 (*n*=1618, 78.20%), and the PHQ-9 (*n*=1626, 78.59%). In those subjects, the three-month PCL-5 total score was 12.92 (*SD*=13.57), the GAD-7 total score was 3.63 (*SD*=4.46), and the PHQ-9 total score was 5.20 (*SD*=5.21), indicating an overall slightly more pronounced psychopathology compared to in the six-months assessments. All subsequent paired Spearman rank correlations were significant ($p<.001$) with large effects (ϱ=0.69–0.73, see Table S2) [20].

Endorsement of Psychopathological Symptoms

After the count data transformation described in the section above, the data indicated that most individuals in the study sample experienced some degree of symptoms of PTSD (64.19%), GAD (66.60%), or MDD (80.14%). Individuals on average experienced 3.32 (*SD*=4.57, *Mdn*=3) out of 20 PTSD symptoms, 2.5 (*SD*=2.46, *Mdn*=2) out of seven GAD symptoms, and 3.25 (*SD*=2.7, *Mdn*=3) out of nine MDD symptoms. Figure S1 depicts the distributions of individual symptoms in the total sample for PTSD (panel A), GAD (panel B), and MDD (panel C). Symptoms of PTSD were experienced by 7.88% (*taking risks*) to 34.85% (*trouble remembering*) of all individuals. GAD symptoms were reported by 23.73% (*feeling afraid*) to 43.89% (*nervous*) of all cases. With regard to MDD, the rate of symptom endorsement varied between 12.76% (*suicidal ideation*) and 64.09% (*feeling tired*).

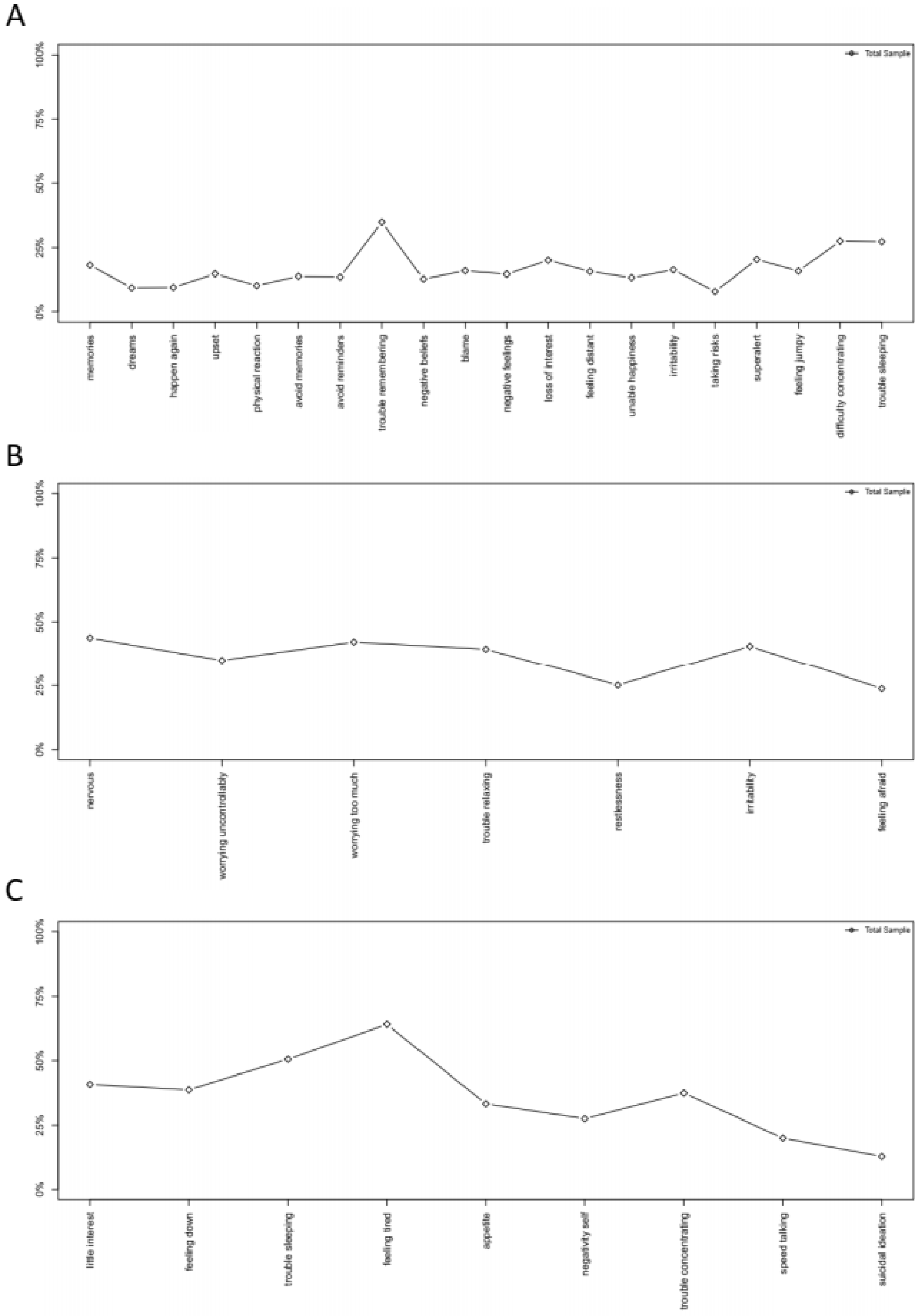

Figure S4. Distribution of individual psychopathological symptoms in the total sample. (A) Post-traumatic Stress Disorder symptoms (PCL-5 item score ≥ 2), (B) Generalized Anxiety Disorder symptoms (GAD-7 item score ≥ 1), (C) Major Depressive Disorder symptoms (PHQ-9 item score ≥ 1). The full list of PROM items is provided in Table S3.

Table S3. Abbreviations and Original Wordings of Items in the PROMs.

PROM	Abbreviation	Wording
PCL-5	*memories*	1. Repeated, disturbing, and unwanted memories of the stressful experience
PCL-5	*dreams*	2. Repeated, disturbing dreams of the stressful experience
PCL-5	*happen again*	3. Suddenly feeling or acting as if the stressful experience were actually happening again (as if you were actually back there reliving it)
PCL-5	*upset*	4. Feeling very upset when something reminded you of the stressful experience
PCL-5	*physical reaction*	5. Having strong physical reactions when something reminded you of the stressful experience (for example, heart pounding, trouble breathing, sweating)
PCL-5	*avoid memories*	6. Avoiding memories, thoughts, or feelings related to the stressful experience
PCL-5	*avoid reminders*	7. Avoiding external reminders of the stressful experience (for example, people, places, conversations, activities, objects, or situations)
PCL-5	*trouble remembering*	8. Trouble remembering important parts of the stressful experience
PCL-5	*negative beliefs*	9. Having strong negative beliefs about yourself, other people, or the world (for example, having thoughts such as: I am bad, there is something seriously wrong with me, no one can be trusted, the world is completely dangerous)
PCL-5	*blame*	10. Blaming yourself or someone else for the stressful experience or what happened after it
PCL-5	*negative feelings*	11. Having strong negative feelings such as fear, horror, anger, guilt, or shame
PCL-5	*loss of interest*	12. Loss of interest in activities that you used to enjoy
PCL-5	*feeling distant*	13. Feeling distant or cut off from other people
PCL-5	*unable happiness*	14. Trouble experiencing positive feelings (for example, being unable to feel happiness or have loving feelings for people close to you)
PCL-5	*irritability*	15. Irritable behavior, angry outbursts, or acting aggressively
PCL-5	*taking risks*	16. Taking too many risks or doing things that could cause you harm
PCL-5	*superalert*	17. Being "superalert" or watchful or on guard
PCL-5	*feeling jumpy*	18. Feeling jumpy or easily startled
PCL-5	*difficulty concentrating*	19. Having difficulty concentrating
PCL-5	*trouble sleeping*	20. Trouble falling or staying asleep
GAD-7	*nervous*	1. Feeling nervous, anxious or on edge
GAD-7	*worrying uncontrollably*	2. Not being able to stop or control worrying
GAD-7	*worrying too much*	3. Worrying too much about different things
GAD-7	*trouble relaxing*	4. Having trouble relaxing
GAD-7	*restlessness*	5. Being so restless that it is hard to sit still
GAD-7	*irritability*	6. Becoming easily annoyed or irritable
GAD-7	*feeling afraid*	7. Feeling afraid, as if something awful might happen
PHQ-9	*little interest*	1. Little interest or pleasure in doing things
PHQ-9	*feeling down*	2. Feeling down, depressed, or hopeless
PHQ-9	*trouble sleeping*	3. Trouble falling or staying asleep, or sleeping too much
PHQ-9	*feeling tired*	4. Feeling tired or having little energy
PHQ-9	*appetite*	5. Poor appetite or overeating

PHQ-9	*negativity self*	6. Feeling bad about yourself — or that you are a failure or have let yourself or your family down
PHQ-9	*trouble concentrating*	7. Trouble concentrating on things, such as reading the newspaper or watching television
PHQ-9	*speed talking*	8. Moving or speaking so slowly that other people could have noticed. Or the opposite — being so fidgety or restless that you have been moving around a lot more than usual
PHQ-9	*suicidal ideation*	9. Thoughts that you would be better off dead or of hurting yourself in some way

Analyses without exclusions

Table S4. Coefficients and effect sizes of the sociodemographic, premorbid, and injury-related factors associated with the PTSD screening diagnosis in the respective logistic regression (LR) model for all participants who completed PCL-5 assessments at six months post TBI (N = 2116).

Factor ǀ Reference	b	SE	z	p	OR	95% CI
age	-0.01	0.01	-1.53	0.13	0.99	0.97–1.00
female ǀ male	0.66	0.67	0.99	0.32	1.94	0.52–7.07
none/primary school ǀ college/university	0.74	0.28	2.66	**0.01**	2.09	1.20–3.58
secondary/high school ǀ college/university	0.24	0.22	1.11	0.27	1.27	0.83–1.94
part-time ǀ full-time employed	0.27	0.29	0.93	0.35	1.31	0.73–2.27
in training ǀ full-time employed	-0.37	0.40	-0.93	0.35	0.69	0.31–1.47
unemployed ǀ full-time employed	0.51	0.30	1.67	0.10	1.66	0.90–2.98
retired ǀ full-time employed	-0.01	0.36	-0.04	0.97	0.99	0.48–2.00
living alone ǀ with someone	-0.27	0.25	-1.07	0.28	0.77	0.46–1.23
premorbid psychiatric history	0.87	0.25	3.53	**<0.001**	2.40	1.46–3.86
road traffic accident ǀ incidental fall	0.57	0.23	2.51	**0.01**	1.77	1.14–2.78
other injury cause ǀ incidental fall	0.92	0.28	3.23	**<0.001**	2.51	1.43–4.37
complicated mild ǀ uncomplicated mild TBI	-0.24	0.25	-0.96	0.34	0.78	0.48–1.28
moderate/severe ǀ uncomplicated mild TBI	-0.93	0.36	-2.59	**0.01**	0.40	0.19–0.80
LOC	0.65	0.29	2.22	**0.03**	1.91	1.10–3.45
extracranial injuries (ISS)	0.00	0.01	-0.07	0.94	1.00	0.98–1.02
admission to ward ǀ emergency room	-0.13	0.29	-0.47	0.64	0.88	0.50–1.54
intensive care unit ǀ emergency room	-0.19	0.39	-0.48	0.63	0.83	0.38–1.79
moderate disability ǀ good recovery	1.34	0.23	5.74	**<0.001**	3.82	2.42–6.06
severe disability ǀ good recovery	1.65	0.33	5.01	**<0.001**	5.20	2.71–9.88
age:female ǀ age:male	0.00	0.01	-0.29	0.77	1.00	0.98–1.02
LOC:female ǀ LOC:male	-0.63	0.44	-1.44	0.15	0.53	0.22–1.26

Note. b = model estimate, SE = standard error, z = z-value, p = p-value, OR = odds ratio, 95% CI = 95% confidence interval (lower, upper bound), TBI = traumatic brain injury, LOC = loss of consciousness, ISS = total injury severity score; bold p-values are significant at $\alpha = 0.05$.

Table S5. Coefficients and effect sizes of the sociodemographic, premorbid, and injury-related factors associated with the GAD screening diagnosis in the respective logistic regression (LR) model for all participants who completed GAD-7 assessments at six months post TBI (N = 2122).

Factor ǀ Reference	b	SE	z	p	OR	95% CI
age	0.00	0.01	-0.04	0.97	1.00	0.98–1.02
female ǀ male	1.33	0.63	2.11	**0.03**	3.78	1.09–13.01
none/primary school ǀ college/university	0.72	0.27	2.67	**0.01**	2.05	1.20–3.46
secondary/high school ǀ college/university	0.37	0.21	1.80	0.07	1.45	0.97–2.16
part-time ǀ full-time employed	-0.22	0.29	-0.75	0.45	0.80	0.44–1.40
in training ǀ full-time employed	-0.25	0.39	-0.64	0.52	0.78	0.35–1.64

unemployed ∣ full-time employed	0.30	0.30	1.02	0.31	1.35	0.75–2.39
retired ∣ full-time employed	-0.17	0.33	-0.51	0.61	0.84	0.44–1.62
living alone ∣ with someone	0.06	0.22	0.27	0.78	1.06	0.68–1.63
premorbid psychiatric history	0.96	0.23	4.25	**<0.001**	2.62	1.67–4.07
road traffic accident ∣ incidental fall	0.63	0.21	3.00	**<0.001**	1.88	1.25–2.85
other injury cause ∣ incidental fall	0.39	0.29	1.32	0.19	1.47	0.82–2.58
complicated mild ∣ uncomplicated mild TBI	0.17	0.25	0.69	0.49	1.19	0.72–1.97
moderate/severe ∣ uncomplicated mild TBI	-0.11	0.34	-0.32	0.75	0.90	0.46–1.74
LOC	0.29	0.27	1.08	0.28	1.34	0.80–2.33
extracranial injuries (ISS)	-0.02	0.01	-2.16	**0.03**	0.98	0.96–1.00
admission to ward ∣ emergency room	-0.27	0.29	-0.92	0.36	0.76	0.43–1.36
intensive care unit ∣ emergency room	0.11	0.38	0.29	0.77	1.12	0.53–2.36
moderate disability ∣ good recovery	1.46	0.23	6.50	**<0.001**	4.32	2.79–6.75
severe disability ∣ good recovery	1.79	0.30	5.95	**<0.001**	5.96	3.30–10.73
age:female ∣ age:male	-0.01	0.01	-1.19	0.23	0.99	0.97–1.01
LOC:female ∣ LOC:male	-0.56	0.40	-1.39	0.16	0.57	0.26–1.26

Note. b = model estimate, SE = standard error, z = z-value, p = p-value, OR = odds ratio, 95% CI = 95% confidence interval (lower, upper bound), TBI = traumatic brain injury, LOC = loss of consciousness, ISS = total injury severity score; bold p-values are significant at $\alpha = 0.05$.

Table S6. Coefficients and effect sizes of the sociodemographic, premorbid, and injury-related factors associated with the MDD screening diagnosis in the respective logistic regression (LR) model for all participants who completed PHQ-9 assessments at six months post TBI (N = 2125).

Factor ∣ Reference	b	SE	z	p	OR	95% CI
age	-0.01	0.01	-0.73	0.47	0.99	0.98–1.01
female ∣ male	1.44	0.53	2.72	**0.01**	4.21	1.49–11.88
none/primary school ∣ college/university	0.69	0.23	2.98	**<0.001**	1.99	1.26–3.11
secondary/high school ∣ college/university	0.19	0.18	1.06	0.29	1.21	0.85–1.71
part-time ∣ full-time employed	-0.12	0.26	-0.45	0.65	0.89	0.53–1.46
in training ∣ full-time employed	0.32	0.31	1.04	0.30	1.38	0.75–2.51
unemployed ∣ full-time employed	0.91	0.26	3.54	**<0.001**	2.49	1.50–4.13
retired ∣ full-time employed	0.20	0.29	0.71	0.48	1.22	0.70–2.14
living alone ∣ with someone	0.38	0.19	2.06	**0.04**	1.47	1.01–2.10
premorbid psychiatric history	0.95	0.21	4.60	**<0.001**	2.58	1.72–3.85
road traffic accident ∣ incidental fall	0.28	0.18	1.57	0.12	1.32	0.93–1.88
other injury cause ∣ incidental fall	0.42	0.24	1.74	0.08	1.53	0.94–2.45
complicated mild ∣ uncomplicated mild TBI	-0.37	0.22	-1.71	0.09	0.69	0.45–1.05
moderate/severe ∣ uncomplicated mild TBI	-0.46	0.29	-1.58	0.11	0.63	0.36–1.12
LOC	0.28	0.23	1.23	0.22	1.33	0.85–2.11
extracranial injuries (ISS)	-0.01	0.01	-1.52	0.13	0.99	0.97–1.00
admission to ward ∣ emergency room	-0.17	0.24	-0.73	0.47	0.84	0.53–1.35
intensive care unit ∣ emergency room	-0.10	0.33	-0.31	0.75	0.90	0.47–1.73
moderate disability ∣ good recovery	1.62	0.20	8.18	**<0.001**	5.04	3.43–7.45
severe disability ∣ good recovery	2.24	0.26	8.56	**<0.001**	9.42	5.65–15.79

age:female ∣ age:male	-0.01	0.01	-1.57	0.12	0.99	0.97–1.00
LOC:female ∣ LOC:male	-0.74	0.34	-2.14	**0.03**	0.48	0.24–0.94

Note. b = model estimate, SE = standard error, z = z-value, p = p-value, OR = odds ratio, 95% CI = 95% confidence interval (lower, upper bound), TBI = traumatic brain injury, LOC = loss of consciousness, ISS = total injury severity score; bold p-values are significant at $\alpha = 0.05$.

Table S7. Coefficients and effect sizes of the sociodemographic, premorbid, and injury-related factors associated with the occurrence and frequency of PTSD symptoms in the respective zero-inflated negative binomial (ZINB) model for all participants who completed PCL-5 assessments at six months post TBI (N = 2116).

Factor ∣ Reference	Zero Part						Count Part					
	**b**	**SE**	**z**	**p**	**OR**	**95% CI**	**b**	**SE**	**z**	**p**	**RR**	**95% CI**
age	-0.01	0.02	-0.49	0.62	0.99	0.96–1.02	-0.01	0.00	-1.63	0.10	0.99	0.99–1.00
female ∣ male	-1.63	1.03	-1.58	0.11	0.20	0.03–1.47	0.39	0.29	1.35	0.18	1.47	0.84–2.58
none/primary school ∣ college/university	-0.50	0.55	-0.91	0.36	0.61	0.21–1.78	0.42	0.13	3.34	<.001	1.53	1.19–1.96
secondary/high school ∣ college/university	-0.65	0.38	-1.73	0.08	0.52	0.25–1.09	0.01	0.09	0.08	0.93	1.01	0.85–1.20
part-time ∣ full-time employed	0.08	0.41	0.20	0.84	1.09	0.49–2.42	-0.01	0.13	-0.11	0.91	0.99	0.77–1.27
in training ∣ full-time employed	-0.80	0.77	-1.04	0.30	0.45	0.10–2.03	-0.27	0.17	-1.65	0.10	0.76	0.55–1.05
unemployed ∣ full-time employed	-1.58	1.56	-1.02	0.31	0.21	0.01–4.36	0.15	0.14	1.07	0.28	1.16	0.88–1.53
retired ∣ full-time employed	-0.50	0.58	-0.86	0.39	0.61	0.20–1.89	-0.07	0.15	-0.45	0.66	0.94	0.70–1.25
living alone ∣ with someone	-0.01	0.39	-0.02	0.99	0.99	0.47–2.12	0.07	0.10	0.70	0.49	1.07	0.88–1.31
premorbid psychiatric history	-1.67	0.88	-1.90	0.06	0.19	0.03–1.05	0.26	0.11	2.29	0.02	1.30	1.04–1.62
road traffic accident ∣ incidental fall	-0.75	0.37	-2.04	**0.04**	0.47	0.23–0.97	0.25	0.09	2.73	0.01	1.28	1.07–1.53
other injury cause ∣ incidental fall	-0.97	0.63	-1.54	0.12	0.38	0.11–1.30	0.27	0.13	2.13	0.03	1.31	1.02–1.69
complicated mild ∣ uncomplicated mild TBI	-0.13	0.40	-0.31	0.75	0.88	0.40–1.94	-0.10	0.11	-0.97	0.33	0.90	0.73–1.11
moderate/severe ∣ uncomplicated mild TBI	-0.02	0.90	-0.02	0.98	0.98	0.17–5.73	-0.38	0.15	-2.56	0.01	0.69	0.51–0.92
LOC	-0.49	0.38	-1.29	0.20	0.61	0.29–1.29	0.19	0.11	1.72	0.08	1.21	0.97–1.50
extracranial injuries (ISS)	-0.01	0.02	-0.43	0.67	0.99	0.94–1.04	0.00	0.00	-0.07	0.95	1.00	0.99–1.01
admission to ward ∣ emergency room	-0.77	0.36	-2.14	**0.03**	0.46	0.23–0.94	-0.19	0.12	-1.52	0.13	0.83	0.65–1.06
intensive care unit ∣ emergency room	-1.00	0.77	-1.30	0.19	0.37	0.08–1.66	-0.12	0.17	-0.70	0.48	0.89	0.64–1.23
moderate disability ∣ good recovery	-2.42	1.26	-1.92	0.06	0.09	0.01–1.06	0.61	0.10	6.08	<.001	1.83	1.51–2.23
severe disability ∣ good recovery	-15.92	1692.90	-0.01	0.99	NA*	NA*	0.62	0.14	4.56	<.001	1.86	1.42–2.42
age:female ∣ age:male	0.03	0.02	1.65	0.10	1.03	0.99–1.06	0.00	0.00	-0.65	0.52	1.00	0.99–1.01
LOC:female ∣ LOC:male	0.55	0.64	0.85	0.39	1.73	0.49–6.06	-0.19	0.18	-1.01	0.31	0.83	0.58–1.19

Note. b = model estimate, SE = standard error, z = z-value, p = p-value, OR = odds ratio, 95% CI = 95% confidence interval (lower bound – upper bound), RR = rate ratios, TBI = traumatic brain injury, LOC = loss of consciousness, ISS = total injury severity score; bold p-values are significant at α = 0.05. *not calculated due to insufficient variability. The zero part estimates the association of factors with the absence of symptoms of PTSD; the count part estimates the association of factors with the average number of symptoms of PTSD.

Table S8. Coefficients and effect sizes of the sociodemographic, premorbid, and injury-related factors associated with the occurrence and frequency of GAD symptoms in the respective zero-inflated negative binomial (ZINB) model for all participants who completed GAD-7 assessments at six months post TBI (N = 2122).

Factor ∣ Reference	Zero Part						Count Part					
	b	SE	z	p	OR	95% CI	b	SE	z	P	RR	95% CI
age	0.02	0.01	2.68	**0.01**	1.02	1.01–1.03	0.00	0.00	1.10	0.27	1.00	1.00–1.01
female ∣ male	0.09	0.51	0.18	0.86	1.09	0.40–2.85	0.34	0.14	2.34	**0.02**	1.40	1.06–1.86
none/primary school ∣ college/university	-0.55	0.23	-2.41	**0.02**	0.58	0.36–0.87	0.15	0.06	2.47	**0.01**	1.16	1.03–1.31
secondary/high school ∣ college/university	0.02	0.16	0.11	0.91	1.02	0.74–1.35	0.04	0.05	0.75	0.45	1.04	0.94–1.14
part-time ∣ full-time employed	0.01	0.22	0.04	0.97	1.01	0.65–1.51	-0.03	0.07	-0.54	0.59	0.97	0.85–1.10
in training ∣ full-time employed	0.20	0.29	0.70	0.48	1.23	0.69–2.12	-0.11	0.09	-1.25	0.21	0.90	0.76–1.06
unemployed ∣ full-time employed	-1.15	0.40	-2.92	**<0.001**	0.32	0.14–0.66	0.00	0.07	0.03	0.97	1.00	0.87–1.15
retired ∣ full-time employed	-0.06	0.24	-0.24	0.81	0.94	0.59–1.46	-0.13	0.08	-1.70	0.09	0.88	0.75–1.02
living alone ∣ with someone	0.02	0.17	0.11	0.92	1.02	0.71–1.39	-0.01	0.05	-0.19	0.85	0.99	0.90–1.10
premorbid psychiatric history	-1.00	0.30	-3.37	**<0.001**	0.37	0.22–0.66	0.17	0.06	3.05	**<0.001**	1.19	1.06–1.32
road traffic accident ∣ incidental fall	-0.16	0.16	-1.02	0.31	0.85	0.62–1.14	0.11	0.05	2.39	**0.02**	1.12	1.02–1.23
other injury cause ∣ incidental fall	0.36	0.21	1.71	0.09	1.43	0.92–2.08	0.16	0.07	2.31	**0.02**	1.17	1.02–1.34
complicated mild ∣ uncomplicated mild TBI	-0.21	0.17	-1.22	0.22	0.81	0.57–1.11	-0.05	0.06	-0.82	0.41	0.95	0.86–1.07
moderate/severe ∣ uncomplicated mild TBI	-0.06	0.27	-0.23	0.82	0.94	0.57–1.60	-0.08	0.08	-1.01	0.31	0.92	0.79–1.08
LOC	0.12	0.18	0.68	0.50	1.13	0.80–1.61	0.00	0.06	0.02	0.98	1.00	0.89–1.12
extracranial injuries (ISS)	0.00	0.01	0.09	0.93	1.00	0.99–1.02	0.00	0.00	-1.60	0.11	1.00	0.99–1.00
admission to ward ∣ emergency room	0.02	0.19	0.08	0.94	1.02	0.69–1.43	-0.02	0.06	-0.38	0.71	0.98	0.86–1.10

intensive care unit ǀ emergency room	-0.21	0.29	-0.72	0.47	0.81	0.44–1.33	0.03	0.09	0.29	0.77	1.03	0.87–1.21
moderate disability ǀ good recovery	-0.77	0.19	-4.13	**<0.001**	0.46	0.31–0.64	0.25	0.05	5.04	**<0.001**	1.28	1.16–1.41
severe disability ǀ good recovery	-1.27	0.33	-3.79	**<0.001**	0.28	0.14–0.50	0.27	0.07	3.81	**<0.001**	1.31	1.14–1.50
age:female ǀ age:male	0.00	0.01	-0.50	0.62	1.00	0.98–1.01	0.00	0.00	-1.21	0.23	1.00	0.99–1.00
LOC:female ǀ LOC:male	-0.54	0.31	-1.72	0.09	0.58	0.32–1.07	-0.19	0.09	-2.12	**0.03**	0.83	0.69–0.99

Note. b = model estimate, SE = standard error, z = z-value, p = p-value, OR = odds ratio, 95% CI = 95% confidence interval (lower bound – upper bound), RR = rate ratios, TBI = traumatic brain injury, LOC = loss of consciousness, ISS = total injury severity score; bold p-values are significant at $\alpha = 0.05$. The zero part estimates the association of factors with the absence of symptoms of GAD; the count part estimates the association of factors with the average number of symptoms of GAD.

Table S9. Coefficients and effect sizes of the sociodemographic, premorbid, and injury-related factors associated with the occurrence and frequency of MDD symptoms in the respective zero-inflated negative binomial (ZINB) model for all participants who completed GAD-7 assessments at six months post TBI (N = 2125).

Factor ǀ Reference	Zero Part						Count Part					
	b	SE	z	p	OR	95% CI	b	SE	z	p	RR	95% CI
age	0.01	0.01	1.03	0.30	1.01	0.99–1.03	0.00	0.00	-1.30	0.19	1.00	0.99–1.00
female ǀ male	-1.14	0.77	-1.49	0.14	0.32	0.07–1.43	0.16	0.14	1.15	0.25	1.18	0.89–1.55
none/primary school ǀ college/university	-0.29	0.30	-0.97	0.33	0.75	0.41–1.35	0.17	0.06	2.85	**<.001**	1.18	1.05–1.33
secondary/high school ǀ college/university	0.16	0.21	0.74	0.46	1.17	0.77–1.77	0.05	0.05	1.01	0.31	1.05	0.96–1.14
part-time ǀ full-time employed	0.08	0.31	0.24	0.81	1.08	0.58–1.99	-0.05	0.06	-0.76	0.45	0.95	0.84–1.08
in training ǀ full-time employed	0.47	0.35	1.32	0.19	1.59	0.80–3.19	0.03	0.08	0.33	0.74	1.03	0.88–1.20
unemployed ǀ full-time employed	-0.55	0.49	-1.14	0.25	0.57	0.22–1.49	0.09	0.07	1.31	0.19	1.09	0.96–1.25
retired ǀ full-time employed	0.03	0.32	0.10	0.92	1.03	0.55–1.93	0.01	0.07	0.14	0.88	1.01	0.88–1.16
living alone ǀ with someone	-0.08	0.23	-0.36	0.72	0.92	0.58–1.45	0.13	0.05	2.81	**0.01**	1.14	1.04–1.25
premorbid psychiatric history	-1.10	0.48	-2.28	**0.02**	0.33	0.13–0.86	0.26	0.05	4.71	**<.001**	1.29	1.16–1.44
road traffic accident ǀ incidental fall	-0.03	0.21	-0.15	0.88	0.97	0.64–1.47	0.08	0.04	1.78	0.07	1.08	0.99–1.18
other injury cause ǀ incidental fall	0.32	0.27	1.18	0.24	1.38	0.81–2.36	0.07	0.06	1.16	0.25	1.08	0.95–1.22
complicated mild ǀ uncomplicated mild TBI	-0.14	0.22	-0.61	0.54	0.87	0.56–1.35	-0.05	0.05	-0.84	0.40	0.96	0.86–1.06

moderate/severe ∣ uncomplicated mild TBI	-0.14	0.39	-0.36	0.72	0.87	0.41–1.87	-0.13	0.07	-1.76	0.08	0.88	0.76–1.01
LOC	0.13	0.24	0.55	0.58	1.14	0.72–1.80	0.03	0.06	0.54	0.59	1.03	0.92–1.15
extracranial injuries (ISS)	-0.01	0.01	-0.85	0.40	0.99	0.96–1.02	0.00	0.00	-1.25	0.21	1.00	0.99–1.00
admission to ward ∣ emergency room	0.07	0.25	0.30	0.77	1.08	0.66–1.74	-0.01	0.06	-0.21	0.84	0.99	0.88–1.11
intensive care unit ∣ emergency room	-0.03	0.39	-0.07	0.94	0.97	0.45–2.11	-0.01	0.08	-0.14	0.89	0.99	0.84–1.16
moderate disability ∣ good recovery	-1.65	0.36	-4.58	**<.001**	0.19	0.09–0.39	0.40	0.05	8.35	**<.001**	1.49	1.36–1.64
severe disability ∣ good recovery	-3.59	2.10	-1.71	0.09	0.03	0.00–1.69	0.52	0.07	7.84	**<.001**	1.68	1.48–1.92
age:female ∣ age:male	0.01	0.01	0.50	0.61	1.01	0.98–1.03	0.00	0.00	-0.44	0.66	1.00	0.99–1.00
LOC:female ∣ LOC:male	0.32	0.49	0.66	0.51	1.38	0.53–3.62	-0.11	0.09	-1.25	0.21	0.90	0.75–1.06

Note. b = model estimate, SE = standard error, z = z-value, p = p-value, OR = odds ratio, 95% CI = 95% confidence interval (lower bound – upper bound), RR = rate ratios, TBI = traumatic brain injury, LOC = loss of consciousness, ISS = total injury severity score; bold p-values are significant at $\alpha = 0.05$. The zero part estimates the association of factors with the absence of symptoms of MDD; the count part estimates the association of factors with the average number of symptoms of MDD.

Table S10. Coefficients and effect sizes of the sociodemographic, premorbid, and injury-related factors associated with the intensity of PCL-5 scores in the respective negative binomial (NB) model for all participants who completed PCL-5 assessments at six months post TBI (N = 2116).

Factor ǀ Reference	b	SE	z	p	RR	95% CI
age	0.00	0.00	-1.27	0.20	1.00	0.99–1.00
female ǀ male	0.51	0.22	2.33	0.02	1.67	1.07–2.62
none/primary school ǀ college/university	0.36	0.10	3.72	<0.001	1.43	1.18–1.73
secondary/high school ǀ college/university	0.08	0.07	1.18	0.24	1.09	0.95–1.25
part-time ǀ full-time employed	-0.06	0.10	-0.56	0.58	0.95	0.78–1.15
in training ǀ full-time employed	-0.15	0.13	-1.19	0.23	0.86	0.67–1.11
unemployed ǀ full-time employed	0.19	0.12	1.56	0.12	1.21	0.96–1.54
retired ǀ full-time employed	0.00	0.11	-0.04	0.97	1.00	0.80–1.23
living alone ǀ with someone	0.07	0.08	0.88	0.38	1.07	0.92–1.25
premorbid psychiatric history	0.36	0.10	3.60	<0.001	1.43	1.18–1.74
road traffic accident ǀ incidental fall	0.28	0.07	4.10	<0.001	1.33	1.16–1.53
other injury cause ǀ incidental fall	0.27	0.10	2.75	0.01	1.31	1.08–1.60
complicated mild ǀ uncomplicated mild TBI	-0.04	0.08	-0.52	0.61	0.96	0.82–1.12
moderate/severe ǀ uncomplicated mild TBI	-0.28	0.12	-2.45	0.01	0.75	0.60–0.95
LOC	0.18	0.08	2.17	0.03	1.20	1.01–1.42
extracranial injuries (ISS)	0.00	0.00	-0.23	0.82	1.00	0.99–1.01
admission to ward ǀ emergency room	0.03	0.09	0.28	0.78	1.03	0.86–1.22
intensive care unit ǀ emergency room	0.12	0.13	0.96	0.34	1.13	0.88–1.46
moderate disability ǀ good recovery	0.64	0.08	8.09	<0.001	1.90	1.63–2.21
severe disability ǀ good recovery	0.68	0.12	5.80	<0.001	1.98	1.58–2.50
age:female ǀ age:male	-0.01	0.00	-1.53	0.13	0.99	0.99–1.00
LOC:female ǀ LOC:male	-0.20	0.14	-1.43	0.15	0.82	0.62–1.08

Note. b = model estimate, SE = standard error, z = z-value, p = p-value, RR = rate ratio, 95% CI = 95% confidence interval (lower bound – upper bound), TBI = traumatic brain injury, LOC = loss of consciousness, ISS = total injury severity score; bold p-values are significant at $\alpha = 0.05$.

Table S11. Coefficients and effect sizes of the sociodemographic, premorbid, and injury-related factors associated with the intensity of GAD-7 scores in the respective negative binomial (NB) model for all participants who completed GAD-7 assessments at six months post TBI (N = 2122).

Factor ǀ Reference	b	SE	z	p	RR	95% CI
age	-0.01	0.00	-1.60	0.11	0.99	0.99–1.00
female ǀ male	0.51	0.25	2.05	**0.04**	1.67	1.01–2.78
none/primary school ǀ college/university	0.46	0.11	4.20	**<0.001**	1.58	1.27–1.97
secondary/high school ǀ college/university	0.08	0.08	0.94	0.35	1.08	0.92–1.26
part-time ǀ full-time employed	-0.07	0.11	-0.63	0.53	0.93	0.75–1.17
in training ǀ full-time employed	-0.28	0.14	-1.96	0.05	0.75	0.57–1.01
unemployed ǀ full-time employed	0.23	0.14	1.67	0.09	1.26	0.97–1.65

retired ǀ full-time employed	-0.06	0.13	-0.51	0.61	0.94	0.73–1.20
living alone ǀ with someone	0.04	0.09	0.49	0.62	1.04	0.88–1.25
premorbid psychiatric history	0.51	0.11	4.71	**<0.001**	1.67	1.35–2.07
road traffic accident ǀ incidental fall	0.25	0.08	3.15	**<0.001**	1.28	1.10–1.50
other injury cause ǀ incidental fall	0.10	0.11	0.86	0.39	1.10	0.88–1.39
complicated mild ǀ uncomplicated mild TBI	0.10	0.09	1.04	0.30	1.10	0.92–1.32
moderate/severe ǀ uncomplicated mild TBI	-0.11	0.13	-0.84	0.40	0.89	0.69–1.16
LOC	0.00	0.10	-0.01	0.99	1.00	0.82–1.21
extracranial injuries (ISS)	-0.01	0.00	-1.85	0.06	0.99	0.99–1.00
admission to ward ǀ emergency room	-0.04	0.10	-0.42	0.67	0.96	0.78–1.17
intensive care unit ǀ emergency room	0.12	0.15	0.82	0.41	1.13	0.84–1.51
moderate disability ǀ good recovery	0.67	0.09	7.41	**<0.001**	1.95	1.64–2.32
severe disability ǀ good recovery	0.86	0.13	6.62	**<0.001**	2.37	1.84–3.07
age:female ǀ age:male	0.00	0.00	-1.15	0.25	1.00	0.99–1.00
LOC:female ǀ LOC:male	-0.07	0.16	-0.41	0.68	0.94	0.68–1.28

Note. b = model estimate, SE = standard error, z = z-value, p = p-value, RR = rate ratio, 95% CI = 95% confidence interval (lower bound – upper bound), TBI = traumatic brain injury, LOC = loss of consciousness, ISS = total injury severity score; bold p-values are significant at $\alpha = 0.05$.

Table S12. Coefficients and effect sizes of the sociodemographic, premorbid, and injury-related factors associated with the intensity of PHQ-9 scores in the respective negative binomial (NB) model for all participants who completed GAD-7 assessments at six months post TBI (N = 2125).

Factor ǀ Reference	b	SE	z	p	RR	95% CI
age	0.00	0.00	-1.80	0.07	1.00	0.99–1.00
female ǀ male	0.54	0.20	2.74	**0.01**	1.72	1.16–2.57
none/primary school ǀ college/university	0.30	0.09	3.52	**<0.001**	1.36	1.14–1.61
secondary/high school ǀ college/university	0.04	0.06	0.55	0.58	1.04	0.91–1.17
part-time ǀ full-time employed	-0.06	0.09	-0.64	0.52	0.94	0.79–1.13
in training ǀ full-time employed	-0.11	0.11	-0.92	0.36	0.90	0.72–1.13
unemployed ǀ full-time employed	0.28	0.11	2.56	**0.01**	1.32	1.07–1.63
retired ǀ full-time employed	0.09	0.10	0.94	0.35	1.10	0.90–1.34
living alone ǀ with someone	0.21	0.07	3.03	**<0.001**	1.23	1.08–1.42
premorbid psychiatric history	0.45	0.09	5.24	**<0.001**	1.57	1.33–1.86
road traffic accident ǀ incidental fall	0.12	0.06	1.86	0.06	1.12	0.99–1.27
other injury cause ǀ incidental fall	0.07	0.09	0.75	0.45	1.07	0.90–1.28
complicated mild ǀ uncomplicated mild TBI	-0.06	0.07	-0.88	0.38	0.94	0.81–1.09
moderate/severe ǀ uncomplicated mild TBI	-0.20	0.11	-1.89	0.06	0.82	0.66–1.01
LOC	0.07	0.08	0.87	0.39	1.07	0.92–1.25
extracranial injuries (ISS)	0.00	0.00	-1.02	0.31	1.00	0.99–1.00
admission to ward ǀ emergency room	-0.01	0.08	-0.18	0.86	0.99	0.84–1.16
intensive care unit ǀ emergency room	0.02	0.12	0.21	0.83	1.02	0.81–1.29
moderate disability ǀ good recovery	0.81	0.07	11.34	**<0.001**	2.24	1.95–2.58
severe disability ǀ good recovery	1.05	0.10	10.26	**<0.001**	2.85	2.34–3.50
age:female ǀ age:male	-0.01	0.00	-1.61	0.11	0.99	0.99–1.00

LOC:female ∣ LOC:male	-0.21	0.13	-1.65	0.10	0.81	0.63–1.04

Note. b = model estimate, SE = standard error, z = z-value, p = p-value, RR = rate ratio, 95% CI = 95% confidence interval (lower bound – upper bound), TBI = traumatic brain injury, LOC = loss of consciousness, ISS = total injury severity score; bold p-values are significant at $\alpha = 0.05$.

References

1. von Steinbuechel, N.; Rauen, K.; Covic, A.; Krenz, U.; Bockhop, F.; Mueller, I.; Cunitz, K.; Polinder, S.; Steyerberg, E.W.; Vester, J.; et al. Sensitivity of Outcome Instruments in a Priori Selected Patient Groups after Traumatic Brain Injury: Results from the CENTER-TBI Study. *PLoS ONE* **2023**, *18*, e0280796, doi:10.1371/journal.pone.0280796.
2. Gardner, R.C.; Dams-O'Connor, K.; Morrissey, M.R.; Manley, G.T. Geriatric Traumatic Brain Injury: Epidemiology, Outcomes, Knowledge Gaps, and Future Directions. *Journal of Neurotrauma* **2018**, *35*, 889–906, doi:10.1089/neu.2017.5371.
3. Demakis, G.J.; Hammond, F.M.; Knotts, A. Prediction of Depression and Anxiety 1 Year After Moderate-Severe Traumatic Brain Injury. *Applied Neuropsychology* **2010**, *17*, 183–189, doi:10.1080/09084282.2010.499752.
4. Yue, J.K.; Levin, H.S.; Suen, C.G.; Morrissey, M.R.; Runyon, S.J.; Winkler, E.A.; Puffer, R.C.; Deng, H.; Robinson, C.K.; Rick, J.W.; et al. Age and Sex-Mediated Differences in Six-Month Outcomes after Mild Traumatic Brain Injury in Young Adults: A TRACK-TBI Study. *Neurological Research* **2019**, *41*, 609–623, doi:10.1080/01616412.2019.1602312.
5. Alway, Y.; McKay, A.; Gould, K.R.; Johnston, L.; Ponsford, J. FACTORS ASSOCIATED WITH POSTTRAUMATIC STRESS DISORDER FOLLOWING MODERATE TO SEVERE TRAUMATIC BRAIN INJURY: A PROSPECTIVE STUDY: Research Article: Predictors of PTSD Following TBI. *Depress Anxiety* **2016**, *33*, 19–26, doi:10.1002/da.22396.
6. Hoofien, D.; Vakil, E.; Gilboa, A.; Donovick, P.J.; Barak, O. Comparison of the Predictive Power of Socio-Economic Variables, Severity of Injury and Age on Long-Term Outcome of Traumatic Brain Injury: Sample-Specific Variables versus Factors as Predictors. *Brain Injury* **2002**, *16*, 9–27, doi:10.1080/02699050110088227.
7. Goldstein, F.C.; Levin, H.S. Cognitive Outcome After Mild and Moderate Traumatic Brain Injury in Older Adults. *Journal of Clinical and Experimental Neuropsychology* **2001**, *23*, 739–753, doi:10.1076/jcen.23.6.739.1028.
8. Whelan-Goodinson, R.; Ponsford, J.L.; Schönberger, M.; Johnston, L. Predictors of Psychiatric Disorders Following Traumatic Brain Injury. *Journal of Head Trauma Rehabilitation* **2010**, *25*, 320–329, doi:10.1097/HTR.0b013e3181c8f8e7.
9. Wang, B.; Zeldovich, M.; Rauen, K.; Wu, Y.-J.; Covic, A.; Muller, I.; Haagsma, J.A.; Polinder, S.; Menon, D.; Asendorf, T.; et al. Longitudinal Analyses of the Reciprocity of Depression and Anxiety after Traumatic Brain Injury and Its Clinical Implications. *JCM* **2021**, *10*, 5597, doi:10.3390/jcm10235597.
10. Hart, T.; Fann, J.R.; Chervoneva, I.; Juengst, S.B.; Rosenthal, J.A.; Krellman, J.W.; Dreer, L.E.; Kroenke, K. Prevalence, Risk Factors, and Correlates of Anxiety at 1 Year After Moderate to Severe Traumatic Brain Injury. *Archives of Physical Medicine and Rehabilitation* **2016**, *97*, 701–707, doi:10.1016/j.apmr.2015.08.436.
11. Bombardier, C.H. Rates of Major Depressive Disorder and Clinical Outcomes Following Traumatic Brain Injury. *JAMA* **2010**, *303*, 1938, doi:10.1001/jama.2010.599.
12. Diaz, A.P.; Schwarzbold, M.L.; Thais, M.E.; Hohl, A.; Bertotti, M.M.; Schmoeller, R.; Nunes, J.C.; Prediger, R.; Linhares, M.N.; Guarnieri, R.; et al. Psychiatric Disorders and Health-Related Quality of Life after Severe Traumatic Brain Injury: A Prospective Study. *Journal of Neurotrauma* **2012**, *29*, 1029–1037, doi:10.1089/neu.2011.2089.
13. Gupte, R.P.; Brooks, W.M.; Vukas, R.R.; Pierce, J.D.; Harris, J.L. Sex Differences in Traumatic Brain Injury: What We Know and What We Should Know. *Journal of Neurotrauma* **2019**, *36*, 3063–3091, doi:10.1089/neu.2018.6171.
14. Iverson, K.M.; Hendricks, A.M.; Kimerling, R.; Krengel, M.; Meterko, M.; Stolzmann, K.L.; Baker, E.; Pogoda, T.K.; Vasterling, J.J.; Lew, H.L. Psychiatric Diagnoses and Neurobehavioral Symptom Severity among OEF/OIF VA Patients with Deployment-Related Traumatic Brain Injury: A Gender Comparison. *Women's Health Issues* **2011**, *21*, S210–S217, doi:10.1016/j.whi.2011.04.019.
15. Bryant, R.A.; Nickerson, A.; Creamer, M.; O'Donnell, M.; Forbes, D.; Galatzer-Levy, I.; McFarlane, A.C.; Silove, D. Trajectory of Post-Traumatic Stress Following Traumatic Injury: 6-Year Follow-Up. *Br J Psychiatry* **2015**, *206*, 417–423, doi:10.1192/bjp.bp.114.145516.
16. Barker-Collo, S.; Theadom, A.; Ameratunga, S.; Jones, K.; Jones, A.; Starkey, N.; Feigin, V.L. Prevalence and Predictors of Post-Traumatic Stress Disorder in Adults One Year Following Traumatic Brain Injury: A Population-Based Study. *Brain Impairment* **2013**, *14*, 425–435, doi:10.1017/BrImp.2013.27.
17. Levin, H.S.; Temkin, N.R.; Barber, J.; Nelson, L.D.; Robertson, C.; Brennan, J.; Stein, M.B.; Yue, J.K.; Giacino, J.T.; McCrea, M.A.; et al. Association of Sex and Age With Mild Traumatic Brain Injury–Related Symptoms: A TRACK-TBI Study. *JAMA Netw Open* **2021**, *4*, e213046, doi:10.1001/jamanetworkopen.2021.3046.

18. Scholten, A.C.; Haagsma, J.A.; Cnossen, M.C.; Olff, M.; van Beeck, E.F.; Polinder, S. Prevalence of and Risk Factors for Anxiety and Depressive Disorders after Traumatic Brain Injury: A Systematic Review. *Journal of Neurotrauma* **2016**, *33*, 1969–1994, doi:10.1089/neu.2015.4252.
19. Nabasny, A.; Myrga, J.M.; Juengst, S.B. Neurobehavioral Symptoms by Gender and Experience of Nightmares after Traumatic Brain Injury. *Rehabilitation Psychology* **2020**, *65*, 186–191, doi:10.1037/rep0000320.
20. Gomez, R.; Skilbeck, C.; Thomas, M.; Slatyer, M. Growth Mixture Modeling of Depression Symptoms Following Traumatic Brain Injury. *Front. Psychol.* **2017**, *8*, 1320, doi:10.3389/fpsyg.2017.01320.
21. Stein, M.B.; Jain, S.; Giacino, J.T.; Levin, H.; Dikmen, S.; Nelson, L.D.; Vassar, M.J.; Okonkwo, D.O.; Diaz-Arrastia, R.; Robertson, C.S.; et al. Risk of Posttraumatic Stress Disorder and Major Depression in Civilian Patients After Mild Traumatic Brain Injury: A TRACK-TBI Study. *JAMA Psychiatry* **2019**, *76*, 249, doi:10.1001/jamapsychiatry.2018.4288.
22. Haarbauer-Krupa, J.; Taylor, C.A.; Yue, J.K.; Winkler, E.A.; Pirracchio, R.; Cooper, S.R.; Burke, J.F.; Stein, M.B.; Manley, G.T.; the TRACK-TBI Investigators Screening for Post-Traumatic Stress Disorder in a Civilian Emergency Department Population with Traumatic Brain Injury. *Journal of Neurotrauma* **2017**, *34*, 50–58, doi:10.1089/neu.2015.4158.
23. DiSanto, D.; Kumar, R.G.; Juengst, S.B.; Hart, T.; O'Neil-Pirozzi, T.M.; Zasler, N.D.; Novack, T.A.; Dillahunt-Aspillaga, C.; Graham, K.M.; Cotner, B.A.; et al. Employment Stability in the First 5 Years After Moderate-to-Severe Traumatic Brain Injury. *Archives of Physical Medicine and Rehabilitation* **2019**, *100*, 412–421, doi:10.1016/j.apmr.2018.06.022.
24. Haagsma, J.A.; Scholten, A.C.; Andriessen, T.M.J.C.; Vos, P.E.; Van Beeck, E.F.; Polinder, S. Impact of Depression and Post-Traumatic Stress Disorder on Functional Outcome and Health-Related Quality of Life of Patients with Mild Traumatic Brain Injury. *Journal of Neurotrauma* **2015**, *32*, 853–862, doi:10.1089/neu.2013.3283.
25. Buckman, J.E.J.; Saunders, R.; Stott, J.; Arundell, L.-L.; O'Driscoll, C.; Davies, M.R.; Eley, T.C.; Hollon, S.D.; Kendrick, T.; Ambler, G.; et al. Role of Age, Gender and Marital Status in Prognosis for Adults with Depression: An Individual Patient Data Meta-Analysis. *Epidemiol Psychiatr Sci* **2021**, *30*, e42, doi:10.1017/S2045796021000342.
26. Paparrigopoulos, T.; Melissaki, A.; Tzavellas, E.; Karaiskos, D.; Ilias, I.; Kokras, N. Increased Co-Morbidity of Depression and Post-Traumatic Stress Disorder Symptoms and Common Risk Factors in Intensive Care Unit Survivors: A Two-Year Follow-up Study. *International Journal of Psychiatry in Clinical Practice* **2014**, *18*, 25–31, doi:10.3109/13651501.2013.855793.
27. Neumann, D.; Juengst, S.B.; Bombardier, C.H.; Finn, J.A.; Miles, S.R.; Zhang, Y.; Kennedy, R.; Rabinowitz, A.R.; Thomas, A.; Dreer, L.E. Anxiety Trajectories the First 10 Years After a Traumatic Brain Injury (TBI): A TBI Model Systems Study. *Archives of Physical Medicine and Rehabilitation* **2022**, *103*, 2105–2113, doi:10.1016/j.apmr.2022.07.002.
28. Bown, D.; Belli, A.; Qureshi, K.; Davies, D.; Toman, E.; Upthegrove, R. Post-Traumatic Stress Disorder and Self-Reported Outcomes after Traumatic Brain Injury in Victims of Assault. *PLoS ONE* **2019**, *14*, e0211684, doi:10.1371/journal.pone.0211684.
29. Van Praag, D.L.G.; Cnossen, M.C.; Polinder, S.; Wilson, L.; Maas, A.I.R. Post-Traumatic Stress Disorder after Civilian Traumatic Brain Injury: A Systematic Review and Meta-Analysis of Prevalence Rates. *Journal of Neurotrauma* **2019**, *36*, 3220–3232, doi:10.1089/neu.2018.5759.
30. Mallya, S.; Sutherland, J.; Pongracic, S.; Mainland, B.; Ornstein, T.J. The Manifestation of Anxiety Disorders after Traumatic Brain Injury: A Review. *Journal of Neurotrauma* **2015**, *32*, 411–421, doi:10.1089/neu.2014.3504.
31. van Reekum, R.; Cohen, T.; Wong, J. Can Traumatic Brain Injury Cause Psychiatric Disorders? *J Neuropsychiatry Clin Neurosci* **2000**, *12*, 316–327, doi:10.1176/jnp.12.3.316.
32. Smirl, J.D.; Jones, K.E.; Copeland, P.; Khatra, O.; Taylor, E.H.; Van Donkelaar, P. Characterizing Symptoms of Traumatic Brain Injury in Survivors of Intimate Partner Violence. *Brain Injury* **2019**, *33*, 1529–1538, doi:10.1080/02699052.2019.1658129.
33. van der Horn, H.J.; Spikman, J.M.; Jacobs, B.; van der Naalt, J. Postconcussive Complaints, Anxiety, and Depression Related to Vocational Outcome in Minor to Severe Traumatic Brain Injury. *Archives of Physical Medicine and Rehabilitation* **2013**, *94*, 867–874, doi:10.1016/j.apmr.2012.11.039.
34. Bryant, R.A. Posttraumatic Stress Disorder and Mild Brain Injury: Controversies, Causes and Consequences. *Journal of Clinical and Experimental Neuropsychology* **2001**, *23*, 718–728, doi:10.1076/jcen.23.6.718.1024.

35. French, L.M.; Lange, R.T.; Iverson, G.L.; Ivins, B.; Marshall, K.; Schwab, K. Influence of Bodily Injuries on Symptom Reporting Following Uncomplicated Mild Traumatic Brain Injury in US Military Service Members. *Journal of Head Trauma Rehabilitation* **2012**, *27*, 63–74, doi:10.1097/HTR.0b013e3182248344.
36. Loignon, A.; Ouellet, M.-C.; Belleville, G. A Systematic Review and Meta-Analysis on PTSD Following TBI Among Military/Veteran and Civilian Populations. *Journal of Head Trauma Rehabilitation* **2020**, *35*, E21–E35, doi:10.1097/HTR.0000000000000514.
37. Levin, H.S.; Brown, S.A.; Song, J.X.; McCauley, S.R.; Boake, C.; Contant, C.F.; Goodman, H.; Kotrla, K.J. Depression and Posttraumatic Stress Disorder at Three Months After Mild to Moderate Traumatic Brain Injury. *Journal of Clinical and Experimental Neuropsychology* **2001**, *23*, 754–769, doi:10.1076/jcen.23.6.754.1021.
38. Carroll, E.L.; Manktelow, A.E.; Outtrim, J.G.; Chatfield, D.; Forsyth, F.; Hutchinson, P.J.A.; Tenovuo, O.; Posti, J.P.; Wilson, L.; Sahakian, B.J.; et al. Influence of Concomitant Extracranial Injury on Functional and Cognitive Recovery From Mild Versus Moderate to Severe Traumatic Brain Injury. *Journal of Head Trauma Rehabilitation* **2020**, *35*, E513–E523, doi:10.1097/HTR.0000000000000575.
39. O'Donnell, M.L.; Creamer, M.; Holmes, A.C.N.; Ellen, S.; McFarlane, A.C.; Judson, R.; Silove, D.; Bryant, R.A. Posttraumatic Stress Disorder After Injury: Does Admission to Intensive Care Unit Increase Risk? *Journal of Trauma: Injury, Infection & Critical Care* **2010**, *69*, 627–632, doi:10.1097/TA.0b013e3181bc0923.
40. Iverson, G.L. Outcome from Mild Traumatic Brain Injury: *Current Opinion in Psychiatry* **2005**, *18*, 301–317, doi:10.1097/01.yco.0000165601.29047.ae.
41. Schönberger, M.; Ponsford, J.; Gould, K.R.; Johnston, L. The Temporal Relationship Between Depression, Anxiety, and Functional Status after Traumatic Brain Injury: A Cross-Lagged Analysis. *J Int Neuropsychol Soc* **2011**, *17*, 781–787, doi:10.1017/S1355617711000701.
42. Ponsford, J.; Draper, K.; Schönberger, M. Functional Outcome 10 Years after Traumatic Brain Injury: Its Relationship with Demographic, Injury Severity, and Cognitive and Emotional Status. *J. Inter. Neuropsych. Soc.* **2008**, *14*, doi:10.1017/S1355617708080272.
43. Perel, P.; Edwards, P.; Wentz, R.; Roberts, I. Systematic Review of Prognostic Models in Traumatic Brain Injury. *BMC Med Inform Decis Mak* **2006**, *6*, 38, doi:10.1186/1472-6947-6-38.
44. Steyerberg, E.W.; Mushkudiani, N.; Perel, P.; Butcher, I.; Lu, J.; McHugh, G.S.; Murray, G.D.; Marmarou, A.; Roberts, I.; Habbema, J.D.F.; et al. Predicting Outcome after Traumatic Brain Injury: Development and International Validation of Prognostic Scores Based on Admission Characteristics. *PLoS Medicine* **2008**, *5*, e165, doi:10.1371/journal.pmed.0050165.
45. Zeldovich, M.; Wu, Y.-J.; Gorbunova, A.; Mikolic, A.; Polinder, S.; Plass, A.; Covic, A.; Asendorf, T.; Andelic, N.; Voormolen, D.; et al. Influence of Sociodemographic, Premorbid, and Injury-Related Factors on Post-Concussion Symptoms after Traumatic Brain Injury. *JCM* **2020**, *9*, 1931, doi:10.3390/jcm9061931.
46. Nagelkerke, N.J.D. A Note on a General Definition of the Coefficient of Determination. *Biometrika* **1991**, *78*, 691–692, doi:10.1093/biomet/78.3.691.
47. Melo, F. Area under the ROC Curve. In *Encyclopedia of Systems Biology*; Dubitzky, W., Wolkenhauer, O., Cho, K.-H., Yokota, H., Eds.; Springer New York: New York, NY, 2013; pp. 38–39 ISBN 978-1-4419-9862-0.
48. Freund, R.J.; Wilson, W.J.; Sa, P. *Regression Analysis*; Elsevier, 2006; ISBN 978-0-08-052297-5.
49. Berk, R.; MacDonald, J.M. Overdispersion and Poisson Regression. *J Quant Criminol* **2008**, *24*, 269–284, doi:10.1007/s10940-008-9048-4.
50. Lindén, A.; Mäntyniemi, S. Using the Negative Binomial Distribution to Model Overdispersion in Ecological Count Data. *Ecology* **2011**, *92*, 1414–1421, doi:10.1890/10-1831.1.
51. Gardner, W.; Mulvey, E.P.; Shaw, E.C. Regression Analyses of Counts and Rates: Poisson, Overdispersed Poisson, and Negative Binomial Models. *Psychological Bulletin* **1995**, *118*, 392–404, doi:10.1037/0033-2909.118.3.392.
52. Blevins, C.A.; Weathers, F.W.; Davis, M.T.; Witte, T.K.; Domino, J.L. The Posttraumatic Stress Disorder Checklist for DSM-5 (PCL-5): Development and Initial Psychometric Evaluation. *JOURNAL OF TRAUMATIC STRESS* **2015**, *28*, 489–498, doi:10.1002/jts.22059.
53. Spitzer, R.L.; Kroenke, K.; Williams, J.B.W.; Löwe, B. A Brief Measure for Assessing Generalized Anxiety Disorder: The GAD-7. *Arch. Intern. Med.* **2006**, *166*, 1092–1097, doi:10.1001/archinte.166.10.1092.
54. Kroenke, K.; Spitzer, R.L.; Williams, J.B.W. The PHQ-9: Validity of a Brief Depression Severity Measure. *J Gen Intern Med* **2001**, *16*, 606–613, doi:10.1046/j.1525-1497.2001.016009606.x.

55. Bovin, M.J.; Marx, B.P.; Weathers, F.W.; Gallagher, M.W.; Rodriguez, P.; Schnurr, P.P.; Keane, T.M. Psychometric Properties of the PTSD Checklist for Diagnostic and Statistical Manual of Mental Disorders–Fifth Edition (PCL-5) in Veterans. *Psychological Assessment* **2016**, *28*, 1379–1391, doi:10.1037/pas0000254.
56. Teymoori, A.; Real, R.; Gorbunova, A.; Haghish, E.F.; Andelic, N.; Wilson, L.; Asendorf, T.; Menon, D.; von Steinbüchel, N. Measurement Invariance of Assessments of Depression (PHQ-9) and Anxiety (GAD-7) across Sex, Strata and Linguistic Backgrounds in a European-Wide Sample of Patients after Traumatic Brain Injury. *Journal of Affective Disorders* **2020**, *262*, 278–285, doi:10.1016/j.jad.2019.10.035.
57. Green, J.A. Too Many Zeros and/or Highly Skewed? A Tutorial on Modelling Health Behaviour as Count Data with Poisson and Negative Binomial Regression. *Health Psychology and Behavioral Medicine* **2021**, *9*, 436–455, doi:10.1080/21642850.2021.1920416.
58. Hinde, J.; Demétrio, C.G.B. Overdispersion: Models and Estimation. *Computational Statistics & Data Analysis* **1998**, *27*, 151–170, doi:10.1016/S0167-9473(98)00007-3.
59. Stein, M.B.; Ursano, R.J.; Campbell-Sills, L.; Colpe, L.J.; Fullerton, C.S.; Heeringa, S.G.; Nock, M.K.; Sampson, N.A.; Schoenbaum, M.; Sun, X.; et al. Prognostic Indicators of Persistent Post-Concussive Symptoms after Deployment-Related Mild Traumatic Brain Injury: A Prospective Longitudinal Study in U.S. Army Soldiers. *Journal of Neurotrauma* **2016**, *33*, 2125–2132, doi:10.1089/neu.2015.4320.
60. Lambert, D. Zero-Inflated Poisson Regression, with an Application to Defects in Manufacturing. *Technometrics* **1992**, *34*, 1, doi:10.2307/1269547.
61. Bulmer, M.G. *Principles of Statistics*; Dover Publications: New York, 1979; ISBN 978-0-486-63760-0.
62. Cohen, J. *Statistical Power Analysis for the Behavioral Sciences*; 2nd ed.; L. Erlbaum Associates: Hillsdale, N.J, 1988; ISBN 978-0-8058-0283-2.
